# Fullerene C_70_‐Encapsulated Tetrathiafulvalene‐Co Porphyrin Covalent Organic Framework: Driving Multistep Charge Transfer to Boost CO_2_ Photoreduction

**DOI:** 10.1002/advs.202505161

**Published:** 2025-05-11

**Authors:** Ying Jiang, Chong Wang, Tianyang Dong, Yupeng Song, Tao Wang, Haibing Che, Hua Sheng, Bo Wu, Chunru Wang

**Affiliations:** ^1^ Beijing National Laboratory for Molecular Sciences Key Laboratory of Molecular Nanostructure and Nanotechnology Institute of Chemistry Chinese Academy of Sciences Beijing 100190 China; ^2^ University of Chinese Academy of Sciences Beijing 100049 China; ^3^ Key Laboratory of Photochemical Conversion and Optoelectronic Materials Technical Institute of Physics and Chemistry Chinese Academy of Sciences Beijing 100190 China; ^4^ College of Chemistry and Life Sciences Chifeng University Chifeng 024000 China; ^5^ Center for Carbon Neutral Chemistry Institute of Chemistry Chinese Academy of Sciences Beijing 100190 China

**Keywords:** covalent organic framework, fullerenes, long‐lived charge‐separated state, multistep charge transfer, photocatalytic CO_2_ reduction

## Abstract

Constructing an efficient charge transfer system can significantly enhance photocatalytic CO_2_ reduction, yet efficient construction strategies remain to be explored. In this work, fullerene C_70_ is encapsulated into the tetrathiafulvalene‐Co porphyrin (TTF‐CoTPP) COF to fabricate an efficient photocatalyst C_70_@COF. Transient absorption (TA) spectra indicate that C_70_ significantly promotes photogenerated charge separation (0.3 ps), subsequently driving multistep charge transfer within the composite system. This process ultimately yields a long‐lived charge‐separated state, TTF^•+^‐CoTPP‐C_70_^•−^ (>5 ns). Density functional theory (DFT) calculation reveals that the encapsulation of C_70_ forms a new electron transfer pathway and reduces the energy barrier for ^*^COOH intermediate formation. The C_70_@COF exhibits a remarkable CO production rate of 4963.24 µmol g h^−1^, a 1.95‐fold enhancement over the pristine COF. This work highlights the potential of fullerene in boosting photocatalytic CO_2_ reduction performance and offers a facile strategy to design novel COF‐based photocatalysts.

## Graphical abstract

The encapsulation of C_70_ into COF has improved the efficiency of photocatalytic CO_2_ reduction significantly. The introduction of C_70_ drives multistep charge transfer and forms a long‐lived charge‐separated state. It also thermodynamically lowers the energy barrier of the rate‐determining step. This work highlights the advantages of fullerene in designing highly efficient COF‐based photocatalysts with multistep charge transfer properties.

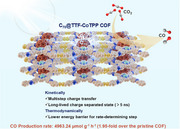

## 1 Introduction

Since the industrial revolution, excessive fossil fuel consumption has led to both energy depletion and excessive CO_2_ emissions.^[^ ^1^, ^2^, ^3^ ^]^ Carbon neutrality has therefore become a global goal essential for promoting eco‐friendly, sustainable development.^[^ ^4^, ^5^ ^]^ Utilization of abundant and clean solar energy to convert CO_2_ into high‐value‐added carbon monoxide (CO, a versatile feedstock for the synthesis of basic organic chemicals and intermediates^[^ ^6^, ^7^ ^]^) is regarded as a promising strategy to address the energy crisis and environmental degradation.^[^ ^8^, ^9^, ^10^ ^]^ Among various photocatalysts, covalent organic frameworks (COFs) have garnered considerable attention for their high porosity, large specific surface area, strong physical adsorption, abundant active sites, and high stability.^[^ ^11^, ^12^ ^]^ However, most COF‐based photocatalysts face the critical scientific challenge of restricted electron delocalization or lack of an oriented electron transmission pathway, resulting in rapid charge recombination and short‐lived charge‐separated states, which limits further improvement of photocatalytic CO_2_ reduction efficiency.^[^ ^13^, ^14^ ^]^ Therefore, achieving rapid separation and further transfer of photogenerated charge within the COF system is urgently required.

Recently, the integration of inorganic semiconductor materials into COFs to form heterojunction structures^[^ ^15^, ^16^, ^17^ ^]^has become a widely adopted approach to enhance photocatalytic CO_2_ reduction performance. However, challenges like energy level mismatches between different components and rapid charge recombination often arise.^[^ ^18^ ^]^ In contrast, the host‐guest strategy offers a simpler construction method with notable charge separation efficiency, making it a highly promising strategy to further improve photocatalytic CO_2_ reduction efficiency.^[^ ^19^ ^]^ Among various guest molecule candidates, fullerene, particularly C_70_, has notable advantages. I) Compared to C_60_, the ellipsoidal structure of C_70_ provides a larger conjugated π system, higher light absorption efficiency, and enhanced electron transport abilities, making it a superior electron confinement agent.^[^ ^20^ ^]^ II) With an appropriate pore size distribution, C_70_ (diameter: 0.78 nm) can be encapsulated into COF pores via geometric host‐guest interactions,^[^ ^21^ ^]^ forming a more stable composite. III) As a classic electron acceptor,^[^ ^22^ ^]^ the low reorganization energy and high electron affinity enable C_70_ to establish a donor‐acceptor (D─A) structure with COF, effectively accepting photogenerated electrons, and achieving efficient electron delocalization and transfer. These characteristics could facilitate electron transfer and inhibit photogenerated charge recombination.^[^ ^23^ ^]^

Porphyrin compounds are renowned for their large conjugated π system, which enables excellent visible light harvesting capability.^[^ ^24^, ^25^ ^]^ Among them, cobalt porphyrin (CoTPP) is commonly employed in photocatalytic CO_2_ reduction due to its metal active sites. During the catalytic process, the tetra‐coordinated cobalt ion at the porphyrin center can activate CO_2_ and insert it into the d‐orbital to form a five‐coordinated cobalt‐CO_2_ adduct that can further generate CO_2_ reduction products.^[^ ^26^ ^]^ Tetrathiafulvalene (TTF) is widely recognized as a prominent electron donor with high electron mobility,^[^ ^27^ ^]^ and it can undergo aromatization in the oxidation process, which can stabilize the radical ion pair, thereby extending the lifetime of the charge‐separated state.^[^ ^28^ ^]^ Notably, the similar oxidation potential of the two monomers enables further charge transfer via hole shift.^[^ ^29^ ^]^ This multistep charge transfer process is similar to the mechanism in natural photosynthesis, facilitating the formation of ultrafast charge separation and long‐lived charge‐separated states,^[^ ^30^, ^31^ ^]^ which is crucial for improving photocatalytic efficiency.

Based on the above properties, CoTPP and TTF were chosen as ligands to construct the TTF‐CoTPP COF model to investigate its photocatalytic performance. Subsequently, a facile physical soaking strategy was employed to encapsulate C_70_ guest molecules into the pores of TTF‐CoTPP COF, fabricating a C_70_@TTF‐CoTPP COF D─A complex (referred to as C_70_@COF). Density functional theory (DFT) calculations revealed that the encapsulation of C_70_ formed a new electron transfer pathway and reduced the energy barrier for ^*^COOH intermediate formation, which is the rate‐determining step in the photocatalytic reduction of CO_2_. Transient absorption (TA) spectroscopy further confirmed a long‐lived charge‐separated state, TTF^•+^‐CoTPP‐C_70_^•−^ (>5 ns), was observed in C_70_@COF through multistep charge transfer. The photocatalytic efficiency was ultimately increased by 1.95 times with the introduction of C_70_, rising from 2551.73 to 4963.24 µmol g h^−1^. This improvement was attributed to the incorporation of C_70_, which triggers multistep charge transfer, ultimately forming a long‐lived charge‐separated state, accompanied by lowering the energy barrier of the rate‐determining step. This strategy highlights a valuable and straightforward approach for designing novel, highly efficient charge‐separated COF‐based photocatalysts with multistep charge transfer properties.

## 2 Results and Discussion

### 2.1 Synthesis and Characterization

**Figure 1:**
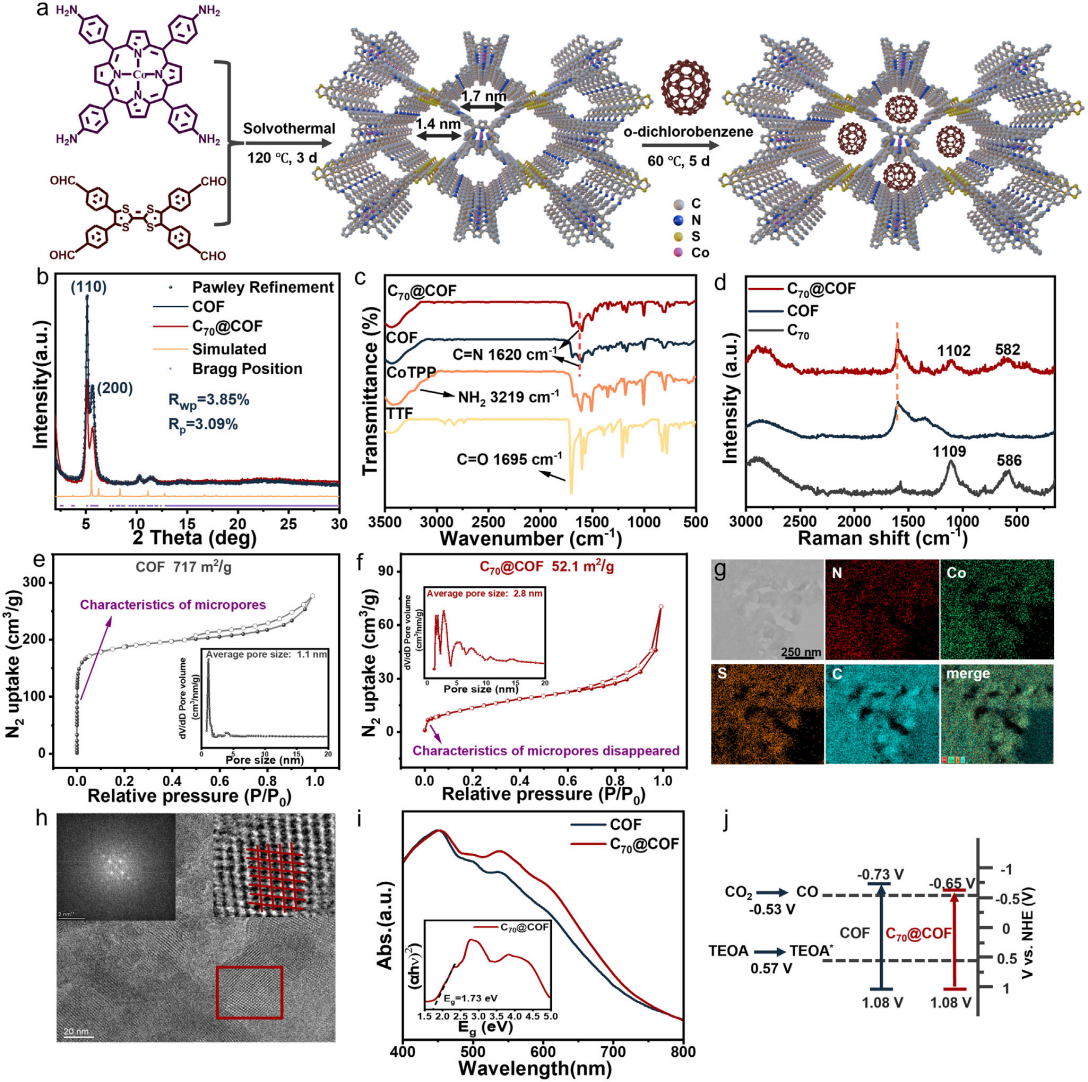
a) Illustration of the synthetic route for TTF‐CoTPP COF and C_70_@COF. b) Experimental and refined PXRD patterns, c) FT‐IR spectra, d) Raman spectra, N_2_ adsorption and desorption isotherms evaluated at 77 K of e) COF, and f) C_70_@COF (inset: pore size distribution profile). g) EDS mapping of C, N, Co, and S elements within a selected area of C_70_@COF. h) Cryo‐TEM image of C_70_@COF (inset: magnified images of highly ordered rhombic pore morphology). i) UV/Vis DRS of C_70_@COF and COF (inset: Tauc plot curve of C_70_@COF, E_g_ = 1.73 eV). j) Band‐structure diagram of C_70_@COF and COF (the reduction potential for CO_2_ to CO: −0.53 V).

The synthesis of TTF‐CoTPP COF was performed following the literature.^[^ ^32^ ^]^ 5,10,15,20‐tetrakis‐(4‐aminophenyl)‐porphyrin‐cobalt(II) (CoTPP) and 2,3,6,7‐tetra(4‐formylphenyl)‐tetrathiafulvalene (TTF‐4CHO) were used as building blocks in a Schiff base reaction within a mixture of benzyl alcohol, *o*‐dichlorobenzene, and 6 m acetic acid at 120 °C. The theoretical simulations were carried out through the Materials Studio software package, revealing the pore sizes of TTF‐CoTPP COF were 1.4 and 1.7 nm, which are suitable for the encapsulation of C_70_. Meanwhile, the spacing between adjacent stacking 2D sheets was determined as 0.32 nm (**Figure** S1, Supporting Information). To obtain C_70_@TTF‐CoTPP COF (referred to as C_70_@COF), the synthesized COF was then immersed in a saturated *o*‐dichlorobenzene solution of C_70_ for seven days (**Figure** **1**a). The crystalline structures were analyzed through Powder X‐ray diffraction (PXRD), followed by structure simulation and Pawley refinement using Materials Studio 2020. As shown in Figure 1b, C_70_@COF exhibited intense diffraction peaks at 5.12° accompanied by a weak peak at 5.7°, which were attributed to the (110) and (200) planes of TTF‐CoTPP COF, respectively.^[^ ^33^ ^]^ The Pawley refinement reproduced the experimentally detected PXRD pattern with a negligible difference (Rp, 3.09% and Rwp, 3.85%), which indicates the correctness of the structure. Importantly, the inclusion of C_70_ had a negligible impact on the crystal structure of TTF‐CoTPP COF. In contrast, TTF‐CoTPP COF demonstrated superior crystallinity, as the crystallinity would slightly decrease during the physical adsorption of C_70_ into the COF pores throughout the soaking process.

Additionally, the chemical structure of the photocatalysts was characterized using Fourier transform infrared (FT‐IR) spectroscopy. The appearance of the characteristic peak at 1620 cm^−1^ signified the successful formation of the C═N bond in the COF framework. This was further supported by the disappearance of the N─H stretching vibration at 3219 cm^−1^ and the C═O stretching vibration peak at 1695 cm^−1^, which belonged to the two monomers.^[^ ^34^ ^]^ Likewise, no significant differences were observed in the FT‐IR spectrum of C_70_@COF (Figure 1c), indicating that the incorporation of C_70_ did not interfere with the successful synthesis of the photocatalyst. Furthermore, Raman spectroscopy (Figure 1d) was employed to further confirm the successful synthesis of the materials. The spectra of C_70_@COF displayed characteristic peaks of both TTF‐CoTPP COF and C_70_, along with a slight shift in the C_70_ peaks, indicating that the charge density has changed after the encapsulation of C_70_ within the COF pores.^[^ ^35^ ^]^

Subsequently, a nitrogen adsorption‐desorption isotherm experiment was performed to further investigate the permanent porosity of the photocatalysts. As shown in Figure 1e, the isotherm profile of TTF‐CoTPP COF exhibited a sharp increase in adsorption at a low relative pressure range, which is characteristic of micropore filling.^[^ ^36^, ^37^ ^]^ The Brunauer‐Emmett‐Teller (BET) surface area of TTF‐CoTPP COF was determined to be 717 m^2^ g^−1^, with a pore size of 1.1 nm, aligning with theoretical calculations and fulfilling the requirements for C_70_ encapsulation. TTF‐CoTPP COF was then soaked in the *o*‐dichlorobenzene solution for seven days in the absence of C_70_. The results showed a decrease in BET surface area to 367.6 m^2^ g^−1^, while the pore size remained unchanged at 1.1 nm (Figure S2, Supporting Information). This reduction in surface area was attributed to the slight reduction of crystallinity during the soaking process. When the COF was soaked with C_70_, it was observed that the adsorption of C_70_ in the pores led to a significant reduction in the specific surface area of C_70_@COF (52.1 m^2^ g^−1^), accompanied by an increase in pore size to 2.8 nm (Figure 1f). The characteristics of micropores disappeared, which was ascribed to the filling of a large number of micropores due to the interaction between C_70_ and COF, leaving behind many mesopores and macropores, thus increasing the average pore size. Additionally, after being thoroughly washed with an *o*‐dichlorobenzene solution to eliminate any adsorbed C_70_ on the surface, C_70_@COF was analyzed. Based on the absorption standard curve of C_70_, the loading amount of C_70_ into the COF pores was calculated to be 2.49 mg (Figure S3, Supporting Information).

The morphological characterization was conducted to give detailed structural information using scanning electron microscopy (SEM) and transmission electron microscopy (TEM). The SEM images revealed that C_70_@COF exhibited a stacked, sheet‐like morphology (Figure S4, Supporting Information). Subsequent energy dispersive X‐ray spectroscopy (EDS) mapping shown in Figure 1g confirmed that the elements N, C, S, and Co were uniformly distributed across the nanosheet and Cryo‐electron microscopy (Cryo‐TEM) further disclosed the sheet‐like morphology and high crystallinity, with the inset magnified image showing the lattice fringe spacing of 1.7 nm, which corresponded to the (110) crystal plane (Figure S5, Supporting Information). Moreover, Figure S6 (Supporting Information) also confirmed the elemental composition of C_70_@COF, and the inset table indicated that the content of cobalt was determined to be 2.61 wt.%. As shown in Figure 1h, the material displayed a highly ordered rhombic pore structure. TTF‐CoTPP COF exhibited a similar morphology with no significant changes (Figure S7, Supporting Information), and the crystallinity remained intact. Furthermore, X‐ray photoelectron spectroscopy (XPS) was employed to analyze the elemental composition and electronic valence state. The XPS survey spectrum confirmed the presence of N, C, S, and Co in C_70_@COF (Figure S8, Supporting Information), which was consistent with the EDS results. In the high‐resolution Co 2p spectrum, the peaks with binding energies of 795.1 and 780 eV can be attributed to Co 2p_1/2_ and Co 2p_3/2_ (Figure S9, Supporting Information), indicating the divalent state of the cobalt ions.^[^ ^38^ ^]^

As shown in Figure 1i, the incorporation of C_70_ resulted in the formation of a D─A structure and broadened the absorption range of C_70_@COF. This slight redshift of the absorption edge was likely due to the enhanced delocalization, which could in turn improve the photocatalytic performance.^[^ ^11^, ^19^ ^]^ Based on the Kubelka‐Munk equation and Tauc‐plot curves, the optical bandgap of C_70_@COF was calculated to be 1.73 eV, narrower than the bandgap of TTF‐CoTPP COF (Figure S10, Supporting Information, 1.81 eV). As depicted in Figures S11 and S12 (Supporting Information), the positive slope of the Mott–Schottky curve indicates that the photocatalysts exhibit n‐type semiconductor characteristics.^[^ ^39^ ^]^ The CB values were calculated as −0.65 and −0.73 V for C_70_@COF and TTF‐CoTPP COF, respectively (vs NHE, pH = 7), which were both more negative than the reduction potential for CO_2_ to CO (theoretically −0.53 V vs NHE, pH 7), demonstrating the thermodynamic feasibility of photocatalytic CO_2_ reduction for both photocatalysts (Figure 1j).

### 2.2 Photocatalytic CO_2_ Reduction Performance

**Figure 2:**
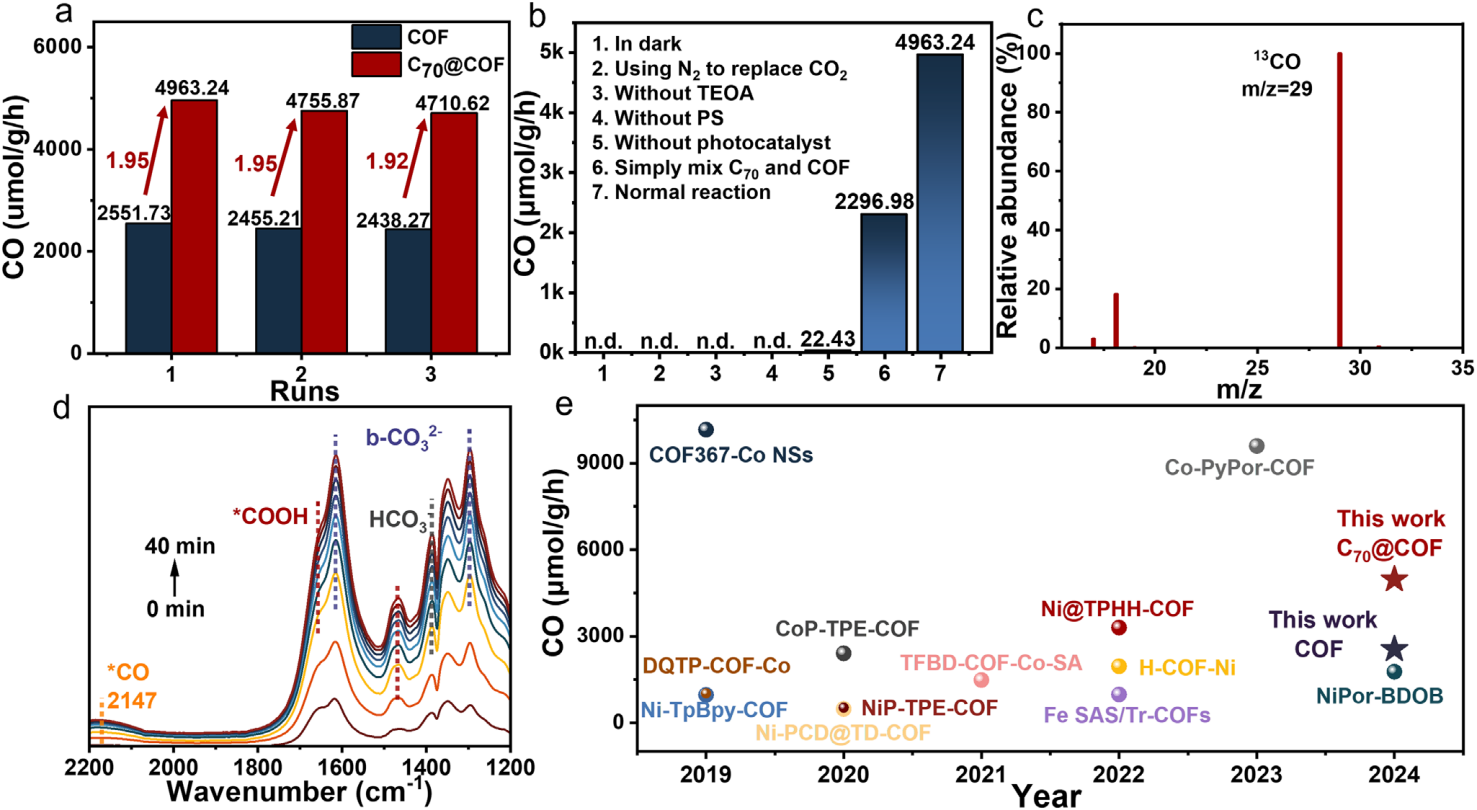
a) Comparison experiment of CO production rate between C_70_@COF and COF over three cycles. b) Control experiments to verify the necessity of light, TEOA, PS, photocatalyst, and CO_2_. c) Isotope labeling experiment via GC‐MS. d). In situ DRIFTS tracking the intermediates during the photocatalytic reduction process. e) Photocatalytic CO_2_ reduction performance of COF‐based photocatalysts in recent years.

The photocatalytic CO_2_ reduction experiment was investigated in a CH_3_CN/H_2_O mixed solution using C_70_@COF or the pristine COF as the photocatalyst, Ru(bpy)_3_Cl_2_·6H_2_O as the photosensitizer (PS), and triethanolamine (TEOA) as the sacrificial agent. As demonstrated in **Figure** **2**a, C_70_@COF achieved a CO production rate of 4963.24 µmol g h^−1^, which is 1.95 times greater than that of TTF‐CoTPP COF (2551.73 µmol g h^−1^) and exceeds most reported COF‐based photocatalysts (Figure 2e; Figures S13–S16, Supporting Information). The apparent quantum yield (AQY) of the CO_2_ photocatalytic process over C_70_@COF was then studied at various wavelengths. As shown in Figure S17 (Supporting Information), the AQY values were positively related to the absorption spectrum, and the highest AQY value was 0.41% at 450 nm. Notably, the efficiency remained stable without significant degradation over three photocatalytic cycles. Likewise, as shown in Figures S18 and S19 (Supporting Information), the XRD results of C_70_@COF after the reaction revealed a slight decrease in crystallinity, while the characteristic peaks remained well‐preserved. TEM images indicated that the photocatalyst maintained its sheet‐like stacked morphology, corroborating its good stability and robustness.

Subsequently, a series of control experiments were carried out to verify the origin of the products obtained fromphotoreduction (Figure 2b). When the reaction was conducted in the dark, no reduction products were detected, suggesting that light irradiation is essential for the process. Similarly, under the same reaction conditions, little or no CO was detected when any of the TEOA, photocatalyst, or PS was absent, or when N_2_ was used instead of CO_2_ as the gas source. These results confirmed that CO production under visible light irradiation occurred only in the presence of the photocatalyst, PS, TEOA, and CO_2_ atmosphere. Interestingly, a simple physical mixture of C_70_ and COF showed a slight decrease in the CO production rate compared to pure COF. This phenomenon further demonstrated that the enhanced photocatalytic activity was a direct consequence of the encapsulation of C_70_ within the COF pores, rather than merely surface interaction.

To identify the carbon source of CO, an isotope labeling experiment was further performed under the same reaction conditions, using ^13^CO_2_ as the substrate instead of CO_2_. As shown in Figure 2c, the peak at m/z = 29 in the GC‐MS corresponds to ^13^CO, indicating that the carbon source of CO in this photocatalytic system originated from CO_2_. In situ diffuse reflectance infrared Fourier transform spectroscopy (DRIFTS) was further employed to explore the intermediates generated during the photocatalytic CO_2_ reduction process. The bicarbonate (HCO_3_^−^ at 1385 cm^−1^) and carbonate species (b‐CO_3_^2−^ at 1294 and 1614 cm^−1^) detected under dark conditions were identified as products formed after CO_2_ adsorption (Figure 2d). As the irradiation time increased, the intensity of these characteristic peaks continued to grow, revealing this was a proton‐assisted photocatalytic reduction process.^[^ ^40^ ^]^ Notably, during 40 min of light irradiation, the characteristic peaks of ^*^CO at 2147 cm^−1^ and ^*^COOH at 1464 and 1656 cm^−1^ were clearly observed, both of which were crucial intermediates in the photocatalytic reduction of CO_2_ to CO.^[^ ^41^ ^]^

### 2.3 Insights into Reaction Mechanism

**Figure 3:**
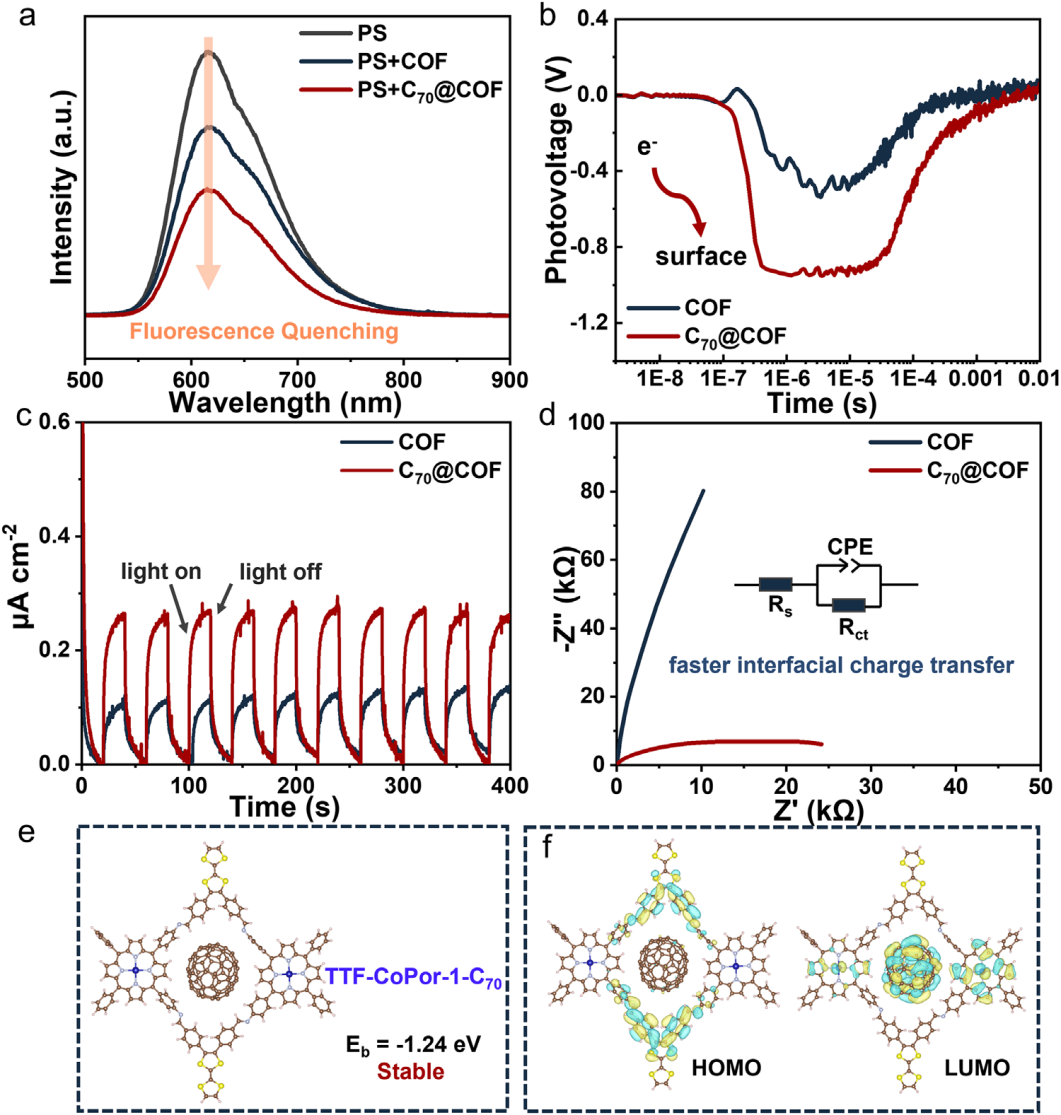
a) Steady‐state PL spectra of photosensitizer (PS), PS+COF, and PS+C_70_@COF. b) TPV response, c) Photocurrent response, d) EIS of COF and C_70_@COF. e) The binding energy for C_70_ is encapsulated within TTF‐CoPor‐1. f) HOMO and LUMO distribution of C_70_@COF.

To clarify the imperative role of C_70_ in boosting photocatalytic efficiency, photoluminescence (PL) and photoelectrochemical analyses were performed to investigate the photogenerated carriers' separation behavior in the composite system. In the steady‐state PL spectra, the fluorescence of PS was quenched after the addition of TTF‐CoTPP COF or C_70_@COF (**Figure** **3**a). This result indicated that both photocatalysts effectively quenched the singlet state of the PS, which can be attributed to the charge transfer process between the PS and photocatalyst.^[^ ^24^, ^42^ ^]^ Importantly, C_70_@COF displayed higher fluorescence quenching ability, suggesting that the encapsulation of C_70_ within the TTF‐CoTPP COF can open up additional and more efficient energy/electron transfer pathways.

Furthermore, the time‐resolved PL decay spectra also confirmed that C_70_@COF exhibited a better fluorescence quenching effect. As shown in Figure S20 (Supporting Information), the fluorescence lifetime of the PS was determined to be 191 ns by fitting with a bi‐exponential function. Upon adding C_70_@COF, the fluorescence lifetime decreased to 169 ns, while it was 185 ns when only TTF‐CoTPP COF was added to the PS. It can be confirmed that the composite system slowed the recombination process of photogenerated carriers. Based on the PL results, it was concluded that the incorporation of C_70_ significantly suppressed charge recombination and promoted photogenerated charge transfer, which likely accounted for the superior photocatalytic performance of C_70_@COF.^[^ ^13^ ^]^

In order to gain a deeper comprehension of the efficiency of photogenerated carriers separation of photocatalysts, surface photovoltage (SPV) and transient photovoltage (TPV) measurements were conducted. As presented in Figure S21 (Supporting Information), the composite material exhibited a stronger negative photovoltage response compared to TTF‐CoTPP COF, further substantiating that rapid charge separation occurred in C_70_@COF under light irradiation and more photogenerated electrons were further efficiently transferred to the surface of the photocatalyst.^[^ ^43^, ^44^ ^]^ The results in TPV spectra were consistent with the SPV spectra, which further indicated that C_70_@COF generated photogenerated carriers with extended lifetimes (Figure 3b). As illustrated in Figure 3c, the photocurrent response demonstrated that the composite material had a significantly higher current density under visible light irradiation, suggesting enhanced photogenerated charge separation. Additionally, the photocurrent response showed highly repeatable characteristics. Electrochemical impedance spectroscopy (EIS) was then employed to assess the charge transfer resistance (Rct). The Nyquist plot revealed that the composite had a smaller semicircle compared to TTF‐CoTPP COF, indicating faster interfacial charge transfer (Figure 3d). Based on the above results, it was confirmed that the encapsulation of C_70_ enabled the composite material to achieve efficient photogenerated carrier separation, thereby optimizing photocatalytic performance.^[^ ^45^ ^]^

Thermodynamic analysis through DFT calculations was further carried out. The binding energy for C_70_ encapsulated within two distinct pores (abbreviated as TTF‐CoPor‐1‐C_70_ and TTF‐CoPor‐2‐C_70_) was calculated to be −1.24 and −0.34 eV, respectively (Figure 3e; Figure S22, Supporting Information). This indicated that the TTF‐CoPor‐1‐C_70_ configuration is more stable, with C_70_ preferentially occupying this pore. All subsequent calculations were based on this structure. As illustrated in Figure S23 (Supporting Information), the highest occupied molecular orbital (HOMO) of TTF‐CoTPP COF was primarily located on the electron donor TTF, while the lowest unoccupied molecular orbital (LUMO) was situated on the CoTPP. Upon the incorporation of C_70_ into the COF pores, the LUMO is predominantly distributed on C_70_ unit, the renowned electron acceptor (Figure 3f). The change of LUMO distribution suggested that the presence of C_70_ might create an additional electron transfer pathway,^[^ ^36^ ^]^ thereby enhancing electron transfer and inhibiting the recombination of photogenerated charges. This result was consistent with the conclusions derived from photoelectrochemical data.

**Figure 4:**
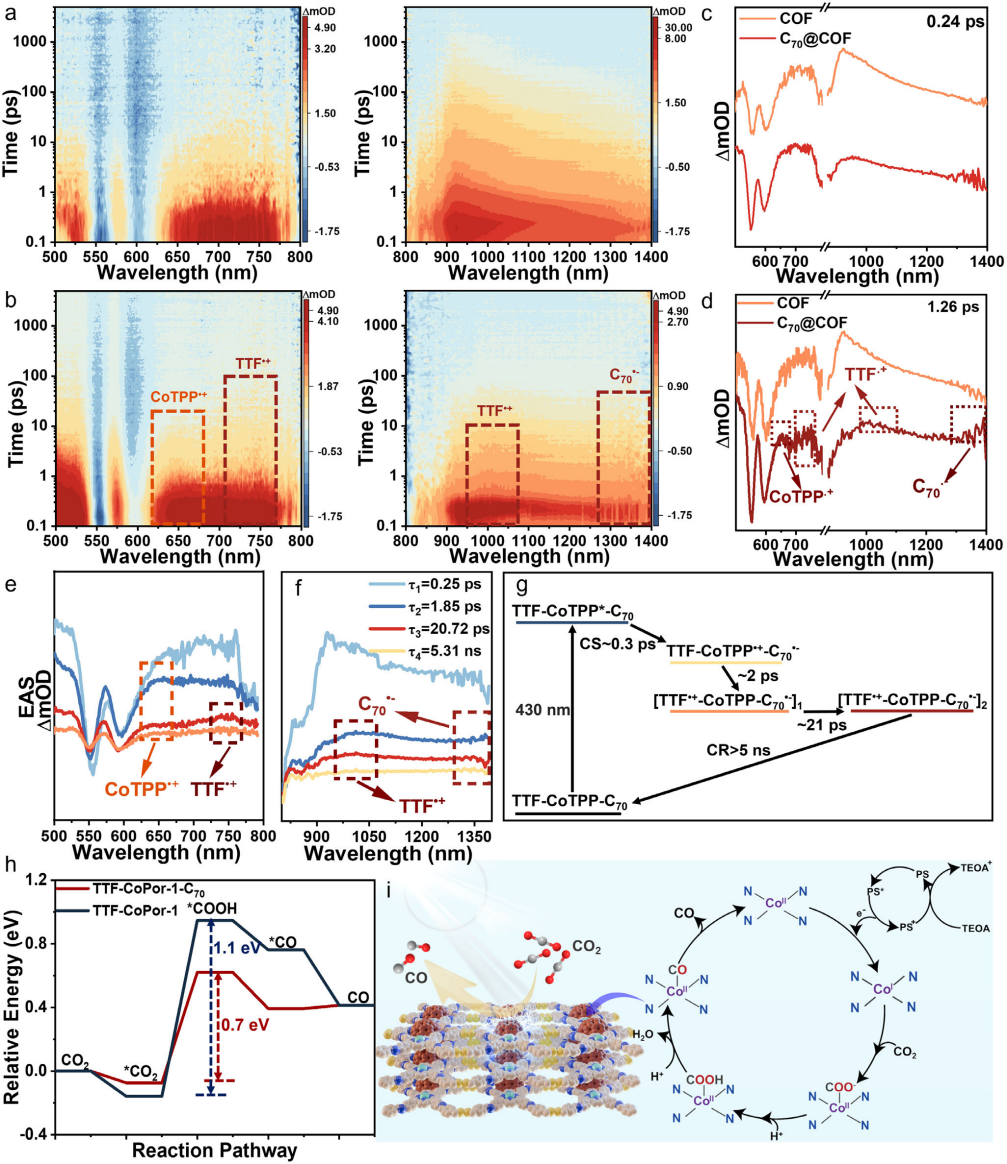
Counter plots of a) COF, and b) C_70_@COF. c) TA spectra at 0.24 ps of COF and C_70_@COF, both of which exhibited the singlet state features. d) TA spectra at 1.26 ps of COF and C_70_@COF. The former had negligible evolution, the latter exhibited CoTPP^•+^, TTF^•+,^ and C_70_^•−^ characteristic absorption bands. Evolution‐associated spectra (EAS) of C_70_@COF with TTF‐^1^CoTPP^*^‐C_70_ (SAS1, baby blue), TTF‐CoTPP^•+^‐C_70_^•−^ (SAS2, blue), [TTF^•+^‐CoTPP‐C_70_^•−^]_1_ (SAS3, red), [TTF^•+^‐CoTPP‐C_70_^•−^]_2_ (SAS4, orange) e) UV region. f) NIR region. g) Simplified schematic kinetic model. h) Calculated Gibbs free energy diagrams for photocatalytic CO_2_ reduction of TTF‐CoPor‐1 and TTF‐CoPor‐1‐C_70_. i) Proposed mechanism of C_70_@COF for photocatalytic CO_2_ reduction.

To investigate electron transfer kinetics within C_70_@COF system, the femtosecond transient absorption spectroscopy (fs‐TAS) was performed. Upon selective excitation of CoTPP at 430 nm, the TA spectra of TTF‐CoTPP COF primarily exhibited localized excited‐state features of CoTPP,^[^ ^46^ ^]^ with negligible spectral evolution (**Figure** **4**a; Figure S24a, Supporting Information). After C_70_ was encapsulated, the characteristic absorptions of CoTPP^•+^ (580 and 650 nm)^[^ ^47^, ^48^, ^49^ ^]^ and C_70_^•−^ (1370 nm)^[^ ^50^ ^]^ were detected within 2 ps upon 430 nm excitation. Interestingly, the evolution from CoTPP^•+^ to TTF^•+^ (750 and 1000 nm)^[^ ^51^ ^]^ was further observed within the following 18 ps, accompanied by the persistent existence of C_70_^•‐^ as shown in Figures 4b and S24b (Supporting Information). These findings indicated that the encapsulation of C_70_ promoted charge separation and triggered a multistep charge transfer process via hole shift from CoTPP to TTF,^[^ ^52^ ^]^ which is expected to generate a long‐lived charge‐separated state.

Given the overlapping excited‐state absorption features of multiple components, the global analysis was employed based on a four‐exponential fitting to resolve each species and obtain evolution‐associated spectra (EAS). As shown in Figure 4e,f, the first resolved component exhibited spectral characteristics similar to COF, corresponding to a locally excited state with a time constant of 0.3 ps (Figure S25, Supporting Information). Then it rapidly evolved into the second component with a time constant of 2 ps, which manifested as the C_70_^•−^ (around 1370 nm) and CoTPP^•+^ (around 580 and 650 nm). Therefore, the simultaneous existence of radical ion pairs indicates that the second component is a charge‐separated state, TTF‐CoTPP^•+^‐C_70_^•−^, which is generated with a time constant of 0.3 ps. The third component retained features of C_70_^•−^ while exhibiting a new absorption at 750 nm assigned to the one‐electron‐oxidized form of TTF meanwhile, along with the decay of CoTPP^•+^ at 650 nm. This indicated the formation of the second charge‐separated state, TTF^•+^‐CoTPP‐C_70_^•−^. Therefore, the evolution from the second to the third component represents a hole shift process from TTF‐CoTPP^•+^‐C_70_^•−^ to TTF^•+^‐CoTPP‐C_70_^•−^. Notably, the global fitting finally resolved a long‐lived fourth component (>5 ns) with spectral features almost identical to the third, albeit with lower intensity, persistent GSB, and an extended lifetime. Transient spectral analysis revealed that encapsulating C_70_ within the COF pores significantly facilitated the separation of excited‐state charges. Furthermore, the hole shift from CoTPP to TTF initiated a multistep charge transfer process. The charge separation occurred with a time constant of 0.3 ps, and the resulting charge‐separated state exhibited a lifetime exceeding 5 ns. A schematic diagram of the excited‐state electron transfer progress in the C_70_@COF system is presented in Figure 4g.

To reveal the underlying mechanism of the excellent photocatalytic CO_2_ reduction activity of C_70_@COF, the intermediate states involved in the photochemical conversion of CO_2_ to CO were outlined in Figure S27 (Supporting Information). Upon the light irradiation, PS was excited, and the photogenerated electrons were transferred to TTF‐CoTPP COF and then confined at C_70_. CO_2_ was then adsorbed at the cobalt active center, followed by a proton‐coupled electron transfer process that generates a carboxyl intermediate (^*^COOH). The intermediate subsequently utilized another electron and proton to produce the ^*^CO intermediate. Finally, CO dissociated from the cobalt active center to yield the final CO product.^[^ ^25^ ^]^ Notably, the formation of the ^*^COOH intermediate is an endothermic process, making it the rate‐determining step in the photocatalytic reduction of CO_2_. According to the Gibbs free energy diagrams presented in Figure 4h, C_70_@COF demonstrated a lower energy barrier for the transition from ^*^CO_2_ to ^*^COOH (1.16 eV) compared to TTF‐CoTPP COF alone (1.30 eV). This indicated that the incorporation of C_70_ effectively reduced the energy barrier to generate the ^*^COOH intermediate, thus facilitating the overall photocatalytic CO_2_ reduction performance. Based on the above results, the proposed mechanism of C_70_@COF for photocatalytic CO_2_ reduction was illustrated in Figure 4i.

## 3 Conclusion

In summary, a C_70_@COF photocatalyst with excellent photocatalytic CO_2_ reduction performance was successfully synthesized by encapsulating C_70_ into TTF‐CoTPP COF pores. The well‐designed C_70_@COF showed an impressive CO formation rate up to 4963.24 µmol g h^−1^, surpassing the performance of the pristine COF (by 1.95 times) and most reported COF‐based photocatalysts. BET tests and spectroscopic analysis were used to confirm the successful encapsulation of C_70_ into the COF pores, where the D─A structure effectively confined electrons within the fullerene. Photoelectrochemical tests indicated that the introduction of C_70_ accelerated charge separation and enhanced transfer efficiency. More importantly, fs‐TA spectroscopy verified that the multistep charge transfer facilitated the formation of a long‐lived charge‐separated state TTF^•+^‐CoTPP‐C_70_^•−^. Thermodynamic analysis through DFT calculations revealed that C_70_@COF also reduced the energy barrier of the rate‐determining step in the CO_2_ reduction process. This work provides a comprehensive analysis of the intrinsic mechanisms behind the boosted CO_2_ photoreduction efficiency, offering valuable insights for the design, development, and modification of efficient photocatalysts.

## Conflict of Interest

The authors declare no conflict of interest.

## Supporting information



Supporting Information

## Data Availability

The data that support the findings of this study are available from the corresponding author upon reasonable request.
