# Supplementary material for: Fullerene C70‐Encapsulated Tetrathiafulvalene‐Co Porphyrin Covalent Organic Framework: Driving Multistep Charge Transfer to Boost CO2 Photoreduction
Source: Adv Sci (Weinh). 2025 May 11;12(28):2505161. doi: 10.1002/advs.202505161 (PMC12302529; doi:10.1002/advs.202505161)
Supplement: Supplementary file 1 — Supporting Information [file ADVS-12-2505161-s001.docx]

Supporting Information

**Fullerene C_70_-Encapsulated Tetrathiafulvalene-Co Porphyrin Covalent Organic Framework: Driving Multistep Charge Transfer to Boost CO_2_ Photoreduction**

*Ying Jiang,^[+][1][2]^ Chong Wang,^[+][1][2]^ Tianyang Dong,^[1][2]^ Yupeng Song,^[2][3]^ Tao Wang,^[1][2]^ Haibing Che,^[4]^ Hua Sheng,^[1][2]^ Bo Wu,*^[1][2][5]^ and Chunru Wang*^[1][2][4]^*

Ying Jiang, Chong Wang, Tianyang Dong, Tao Wang, Hua Sheng, Bo Wu, and Chunru Wang

^1^Beijing National Laboratory for Molecular Sciences, Key Laboratory of Molecular Nanostructure and Nanotechnology, Institute of Chemistry, Chinese Academy of Sciences

^+^These authors contributed equally to this work.

^2^University of Chinese Academy of Sciences

E-mail: zkywubo@iccas.ac.cn; crwang@iccas.ac.cn

Yupeng Song

^2^University of Chinese Academy of Sciences

^3^Key Laboratory of Photochemical Conversion and Optoelectronic Materials, Technical Institute of Physics and Chemistry, Chinese Academy of Sciences

Haibing Che and Chunru Wang

^4^College of Chemistry and Life Sciences, Chifeng University

Bo Wu

^5^Center for Carbon Neutral Chemistry, Institute of Chemistry, Chinese Academy of Sciences

Table of Contents

[Table of Contents 2](#_Toc195706350)

[1. General methods 3](#_Toc195706351)

[2. Characterizations 5](#_Toc195706352)

[3. Quantitative standard curve 9](#_Toc195706353)

[4. Photocatalytic efficiency 9](#_Toc195706354)

[5. Characterizations of photocatalyst after the reaction 11](#_Toc195706355)

[6. Time-resolved PL decay spectra 12](#_Toc195706356)

[7. Surface photovoltage response spectra 12](#_Toc195706357)

[8. DFT Calculation 12](#_Toc195706358)

[9. Transient Absorption Spectra 13](#_Toc195706359)

[10. The intermediate states 14](#_Toc195706360)

[References 15](#_Toc195706361)

1. General methods

(1) Preparation of TTF-CoTPP COF.

The synthesis of TTF-CoTPP COF was conducted based on the method described in previous literature. The detailed procedure was as follows: CoTPP (14.7 mg, 0.02 mmol) and TTF-4CHO (14.97 mg) were added to a Pyrex tube, and subsequently dispersed in a mixture of benzyl alcohol, o-dichlorobenzene, and 6 M acetic acid (3:1:1 v:v, 6 mL), then sonicated for 15 minutes. The tube was frozen at 77 K (liquid N_2_ bath), and after one freeze-pump-thaw cycle. The reaction system was heated at 120°C for three days, then cooled to room temperature and washed with tetrahydrofuran, DMF, and acetone (50 mL× 3) to obtain a dark purple sample. Finally, the product underwent evacuation at 150°C under dynamic vacuum overnight to yield the activated sample.

(2) Preparation of C_70_@TTF-CoTPP COF.

30 mg TTF-CoTPP COF was added to a saturated C_70_ solution in 5 mL of o-dichlorobenzene and sonicated for 15 minutes. The sample vial was then placed in a preheated oven at 60°C and left undisturbed for 7 days. After that, the sample vial was cooled to room temperature, and the obtained C_70_@TTF-CoTPP COF sample was filtered and washed thoroughly with o-dichlorobenzene solution to eliminate any adsorbed C_70_ on the surface. The solid was then dried in a vacuum oven at 60°C for 12 h.

(3) Photocatalytic reduction of CO_2_.

The photocatalytic reduction of CO_2_ experiment was performed in a 400 mL quartz reactor, with an effective volume of 250 mL, under constant stirring. The reaction temperature was controlled at 20°C using a circulating cooling water system. Specifically, 5 mg of photocatalyst, 30 mg of Ru(bpy)_3_Cl_2_·6H_2_O, and a mixed solution of acetonitrile, TEOA, and H_2_O (3:1:1, 15 mL) were sonicated evenly and then added to the quartz reactor. High-purity CO_2_ was purged through the system for 30 minutes to ensure the complete removal of air. A 300W xenon lamp (light intensity: 400 mW cm^-2^; CEL-HXF300-T10, Beijing China Education Au-light Technology Co., Ltd.) equipped with a UV cut-off filter (λ > 420 nm) was used as the light source. After each hour of reaction, 1 mL of the headspace was injected into the gas chromatography (GC 7920, 8890 GC System, Agilent, USA, argon as a carrier gas) to analyze the gaseous products. CO was determined using a flame ionization detector (FID). Control experiments were performed in the absence of CO_2_, light irradiation, photocatalyst or photosensitizer, respectively.

(4) Stability test.

After 4 hours of reaction, the light source was turned off, and the photocatalyst was retrieved by centrifugation. The collected photocatalyst was then washed thoroughly with acetonitrile and dried at 60°C. Subsequently, the experimental steps for the photocatalytic reduction of CO_2_ were repeated.

Furthermore, the apparent quantum yield (AQY) of C_70_@COF was measured by different monochromatic light filters (including 420 nm, 450 nm, 500 nm and 550 nm) under the same condition. Average intensity per unit area was determined using a radiometer (CEL-NP2000-2A, CeAulight, Beijing, China), followed by AQY calculation using the following Equation 1:

$$AQY\left( \% \right)=\frac{2\times the number of evolved CO molecues}{incidented photon number}\times100\% (1)$$

(5) ^13^CO_2_ Isotope Labelling Experiment.

The ^13^CO_2_ Isotope Labelling Experiment was performed under the same reaction conditions, using ^13^CO_2_ as the substrate instead of CO_2_. Specifically, 5 mg of photocatalyst, 30 mg of Ru(bpy)_3_Cl_2_·6H_2_O, and a mixed solution of acetonitrile, TEOA, and H_2_O (3:1:1, 15 mL) were sonicated evenly and then added to the quartz reactor. High-purity ^13^CO_2_ was purged through the system for 15 minutes to ensure the complete removal of air. A 300W xenon lamp (light intensity: 400 mW cm^-2^) equipped with a UV cut-off filter (λ > 420 nm) was used as the light source. After an hour of reaction, 1 mL of the headspace was injected into the gas chromatograph-mass spectrometer (5977B GC/MSD, Agilent) to analyze the gaseous products. ^13^CO was determined using a flame ionization detector (FID).

(6) Density functional theory (DFT) calculation.

DFT ﻿simulations were performed using the freely available CP2K/Quickstep package^[1]^. The 1s electron of H, 2s, 2p electrons of C, O, 3d, 4s electrons of CO and were treated as valence, and the rest core electrons were represented by Goedecker-Teter-Hutter (GTH)^[2,3]^ pseudopotentials. ﻿The Gaussian basis set was double-$\zeta$with one set of polarization functions (DZVP-MOLOPT-SR-GTH)^[4]^, and the plane wave cutoff was set to 400 Ry. The Perdew–Burke–Ernzerhof (PBE)^[5]^ density functional ﻿with the Grimme D3 dispersion correction^[6]^ was used. ﻿The geometries were optimized by Broyden-Fletcher-Goldfarb-Shanno (BFGS) minimizer.

(7) Characterization methods.

Powder X-ray diffraction (PXRD) was used to analyze crystal structure with Miniflex 600 diffractometer using Cu Κα radiation. X-ray photoelectron spectroscopy (XPS) measurements were performed on an ESCALAB 250Xi (Thermo Fisher). Fourier transform infrared (FT-IR) spectra were recorded using KBr on a PerkinElmer Spectrum One. The morphology and structure of the as-prepared photocatalysts were observed by a scanning electron microscope (SEM) (JSM-6700F JEOL, Japan), transmission electron microscopy (TEM) (JEM-2100F, JEOL, Japan), and Cryo-transmission electron microscope (Themis 300). The UV-Vis diffused reflectance spectroscopy (UV-Vis DRS) of powders was carried out using a UV-vis spectrophotometer (UV-3100, Shimadzu Inc., Japan) with BaSO_4_ as the reference. The steady-state photoluminescence (PL) spectra were recorded using HORIBA Fluorescence spectrophotometer and time-resolved PL decay spectra were obtained from FLS980. Nitrogen adsorption-desorption isotherms were collected at 77 K by Micromeritics ASAP 2460 instrument. The samples were degassed at 120 °C overnight before test and the BET surface areas were determined from the amount of N_2_ adsorbed via the Brunauer-Emmett-Teller (BET) equilibrium equation.

2. Characterizations

**
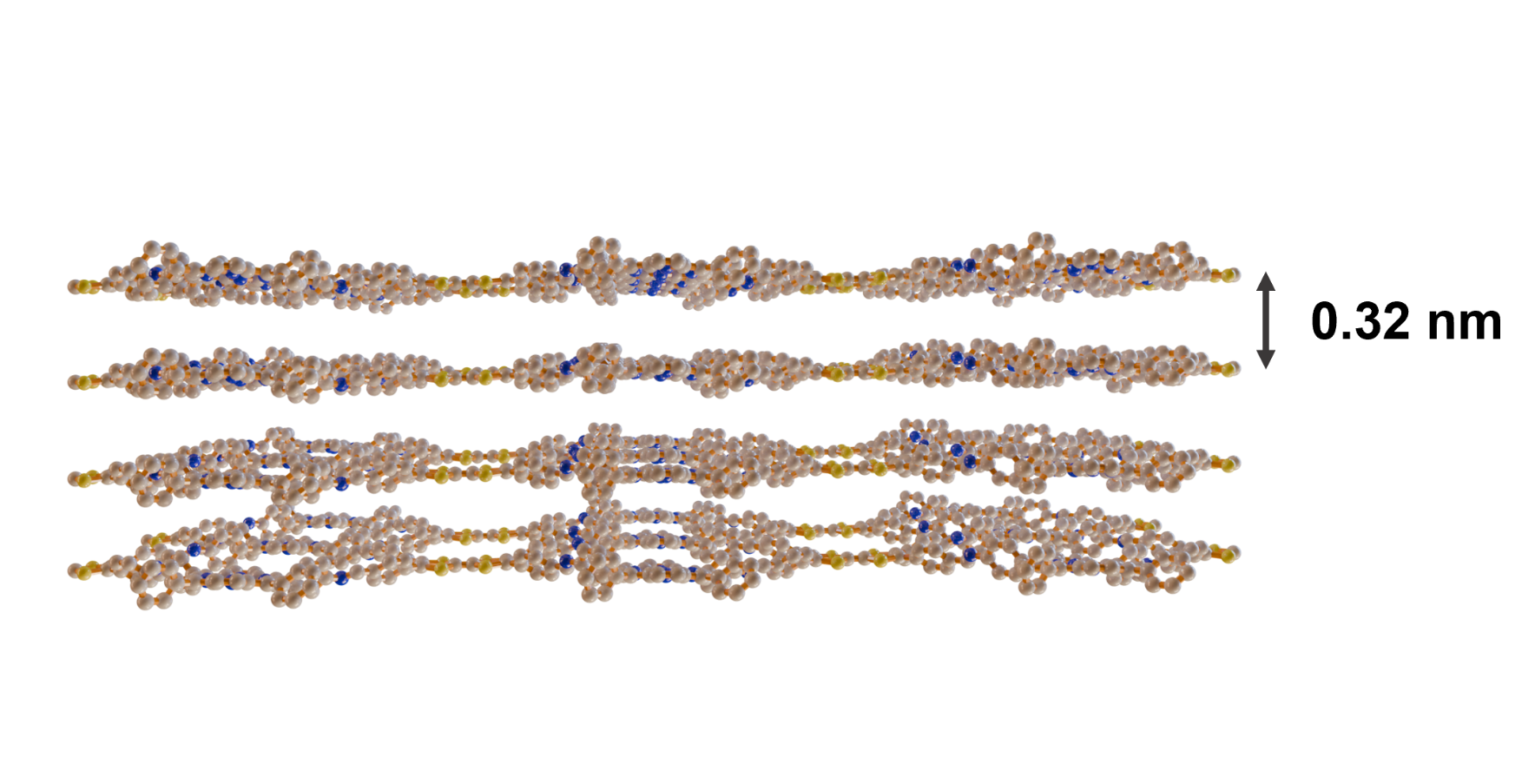
**

**Figure S1.** Interlayer spacing of TTF-CoTPP COF.

**
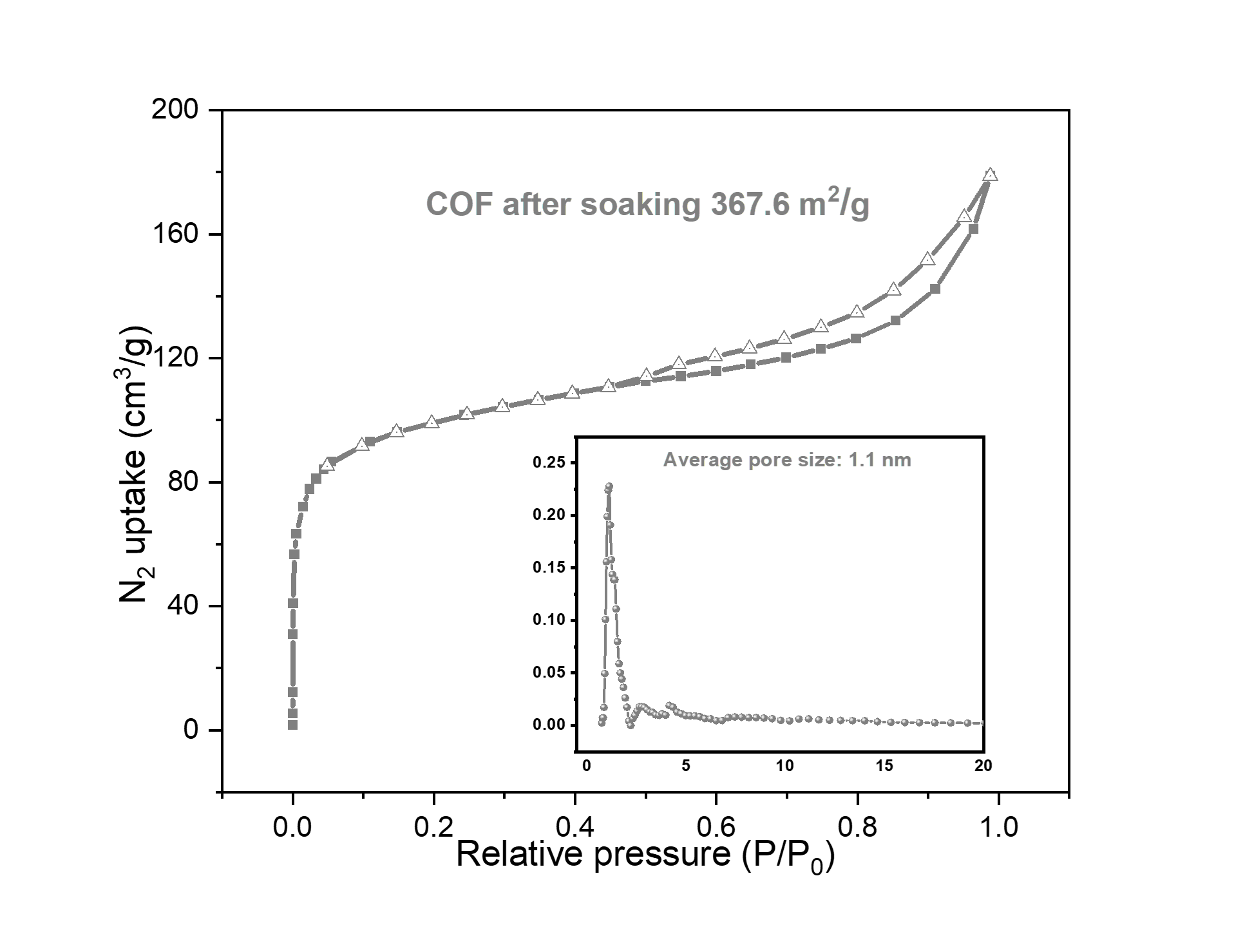
**

**Figure S2.** N_2_ adsorption-desorption isotherm of TTF-CoTPP COF after soaking in *o*-Dichlorobenzene, inset was the pore size distribution profile.


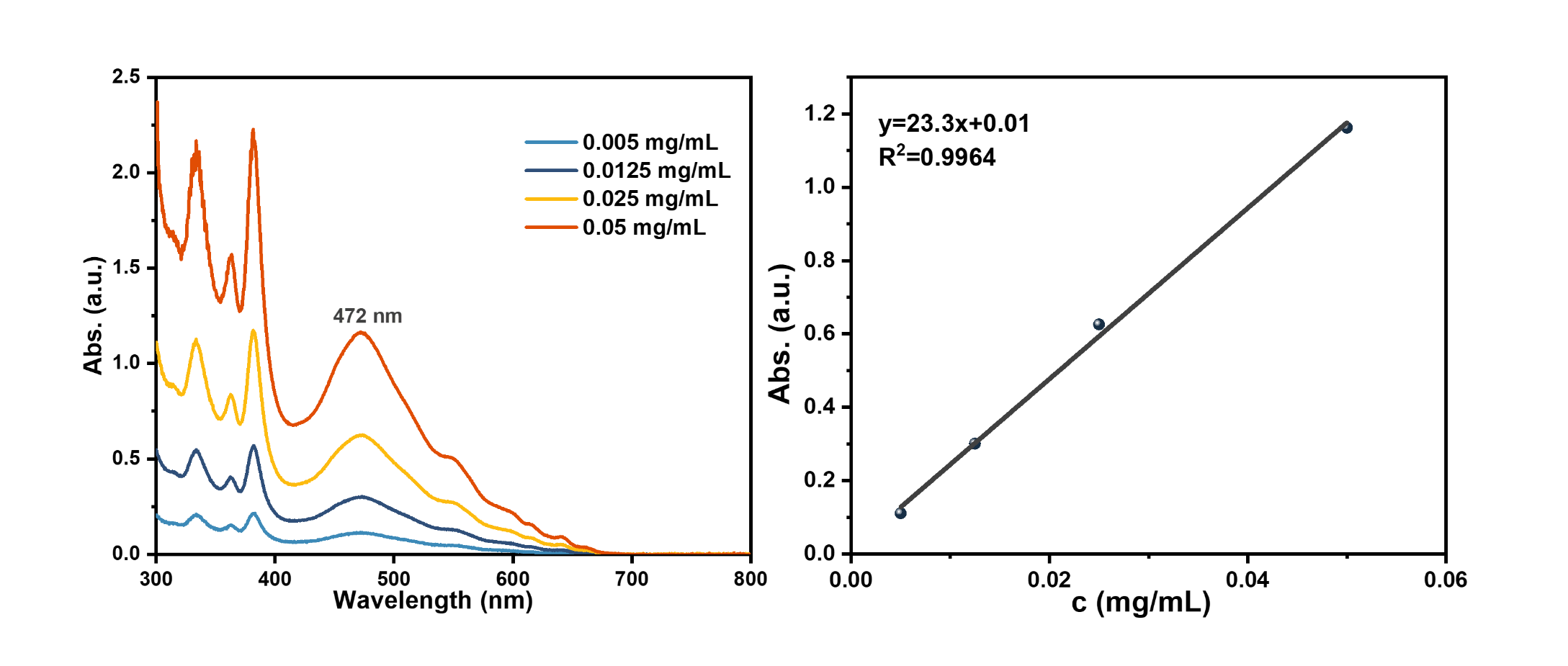


**Figure S3.** The UV-Vis absorption spectra of C_70_ at different concentrations were used to establish a standard curve with the characteristic peak at 473 nm.

After soaking for one week, the supernatant was diluted 200 times and tested using a UV-Vis spectrophotometer to determine the remaining C_70_ content, and the loading amount was calculated to be 16.6%.


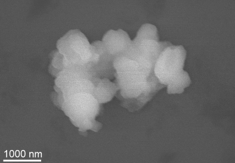


**Figure S4.** SEM images of C_70_@COF.


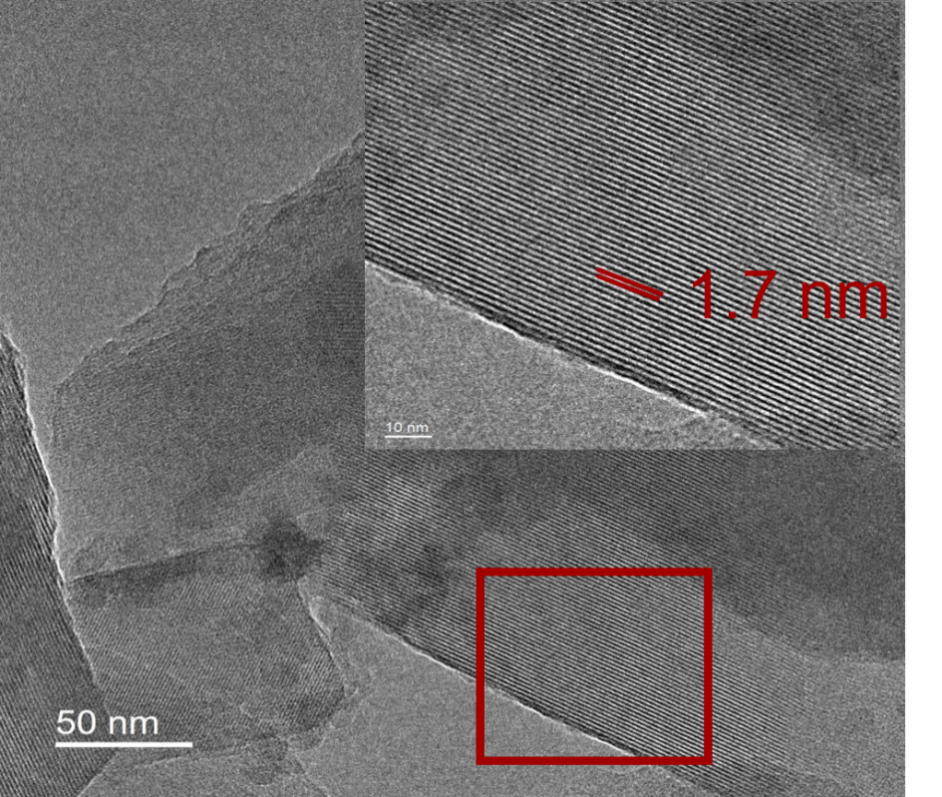


**Figure S5.** Cryo-TEM image with an inset of the magnified lattice structure of C_70_@COF.


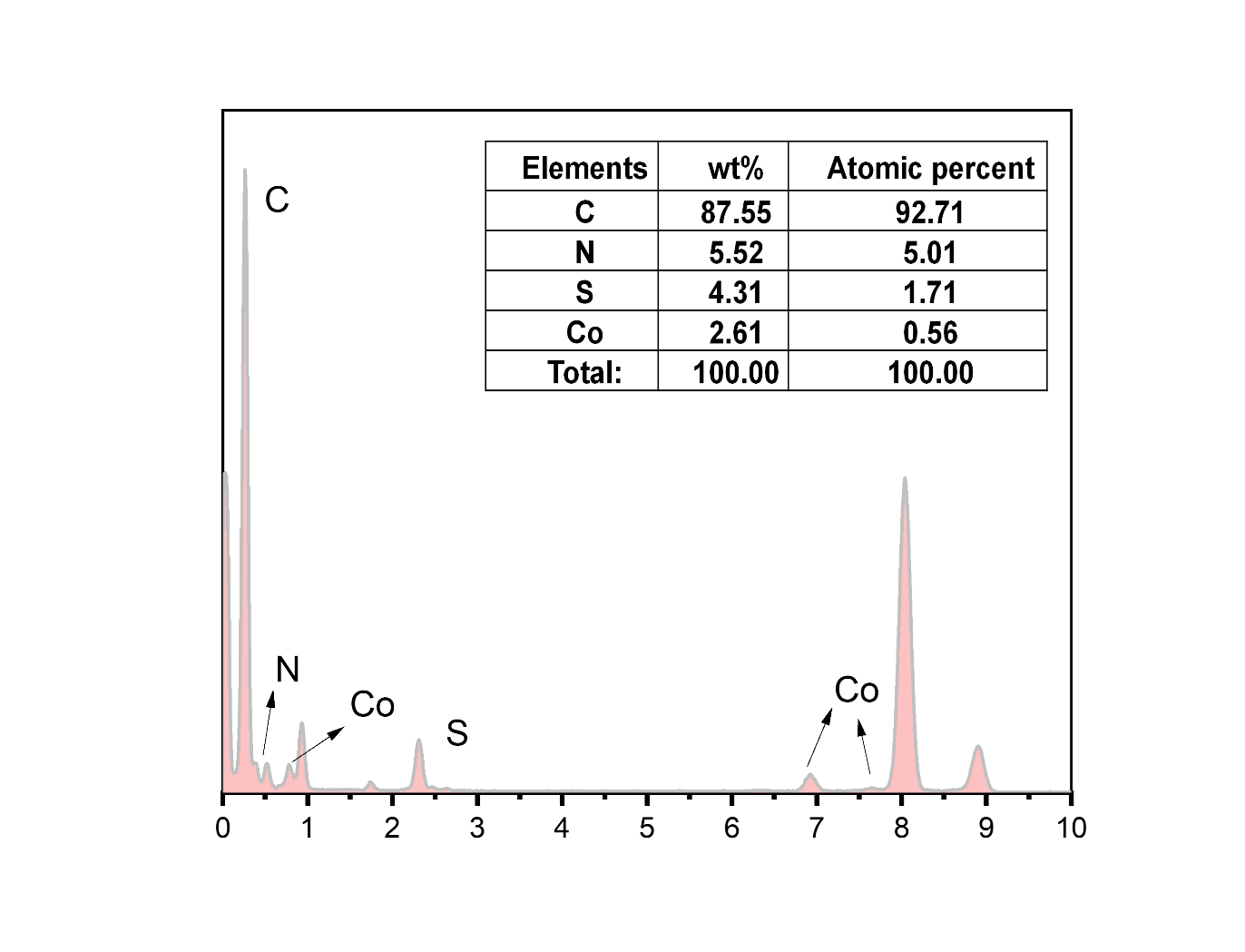


**Figure S6.** Total EDS Mapping Spectrum of C_70_@COF, inset was the element content table.


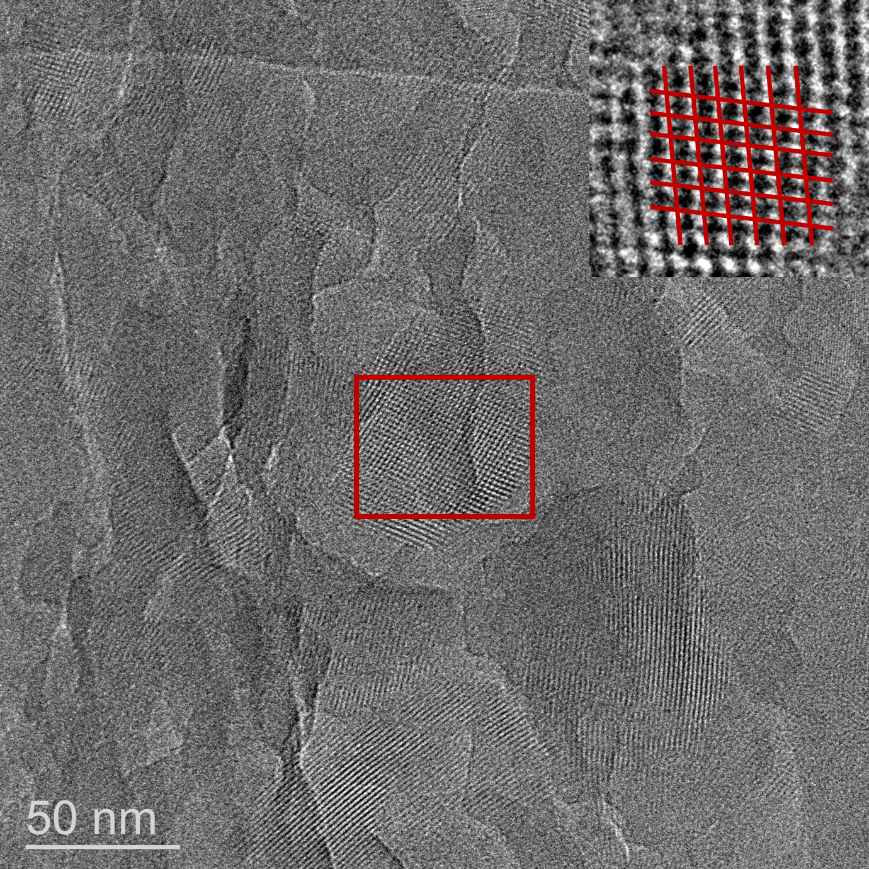


**Figure S7.** TEM Image of TTF-CoTPP COF, inset was the magnified pores.


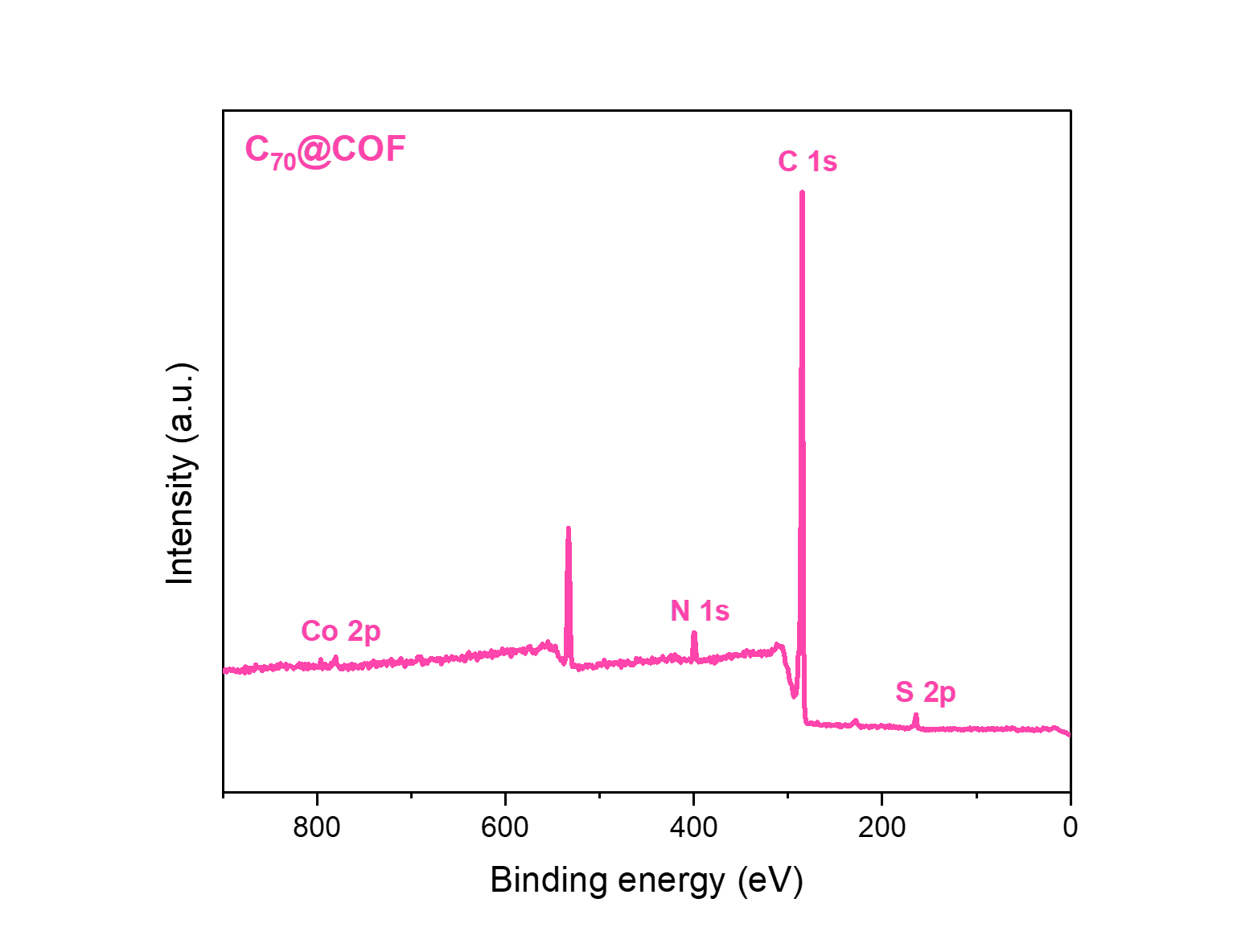


**Figure S8.** XPS Survey Spectra of C_70_@COF.


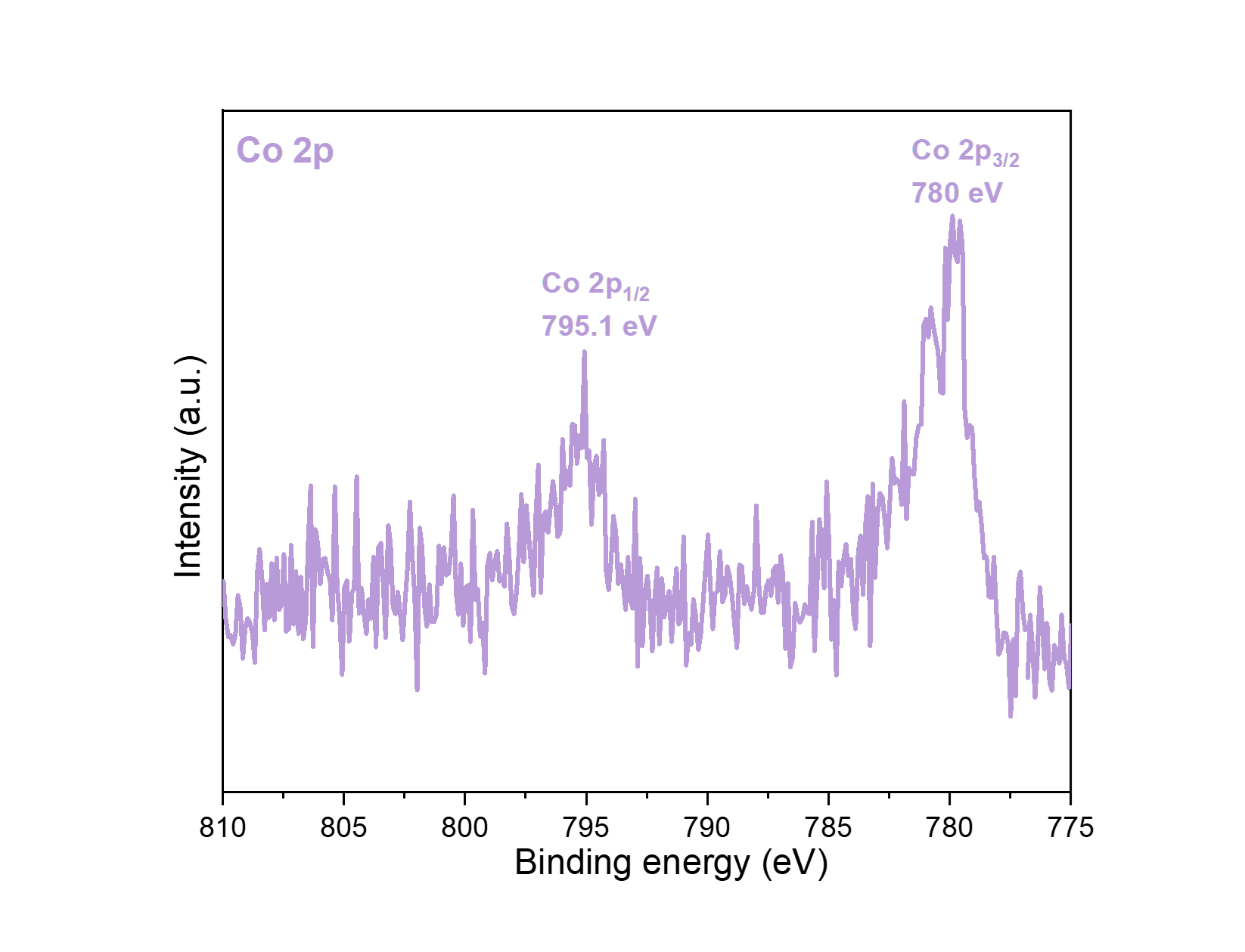


**Figure S9.** High-resolution XPS spectra of Co 2p for C_70_@COF.


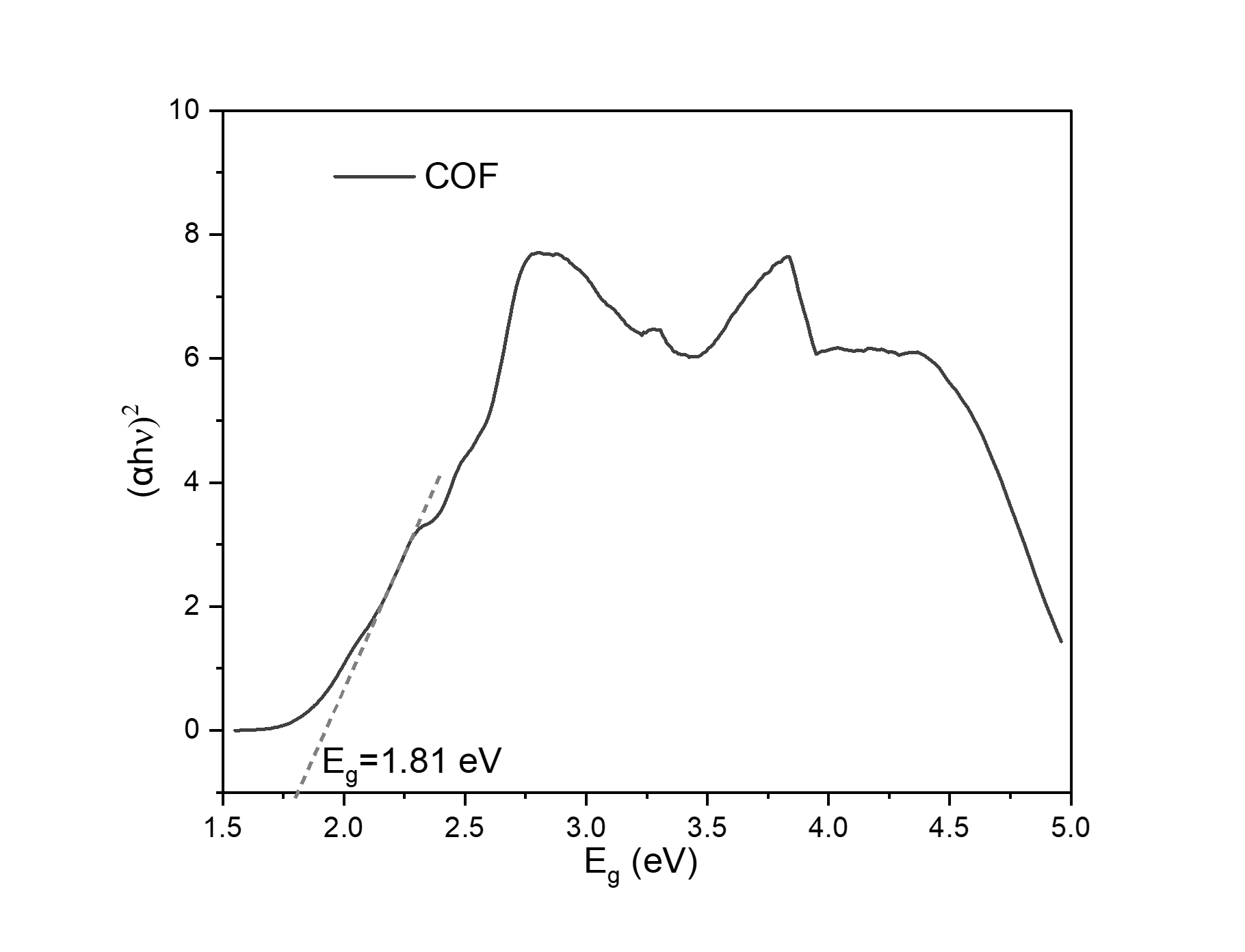


**Figure S10.** Tauc Plot curve of TTF-CoTPP COF.


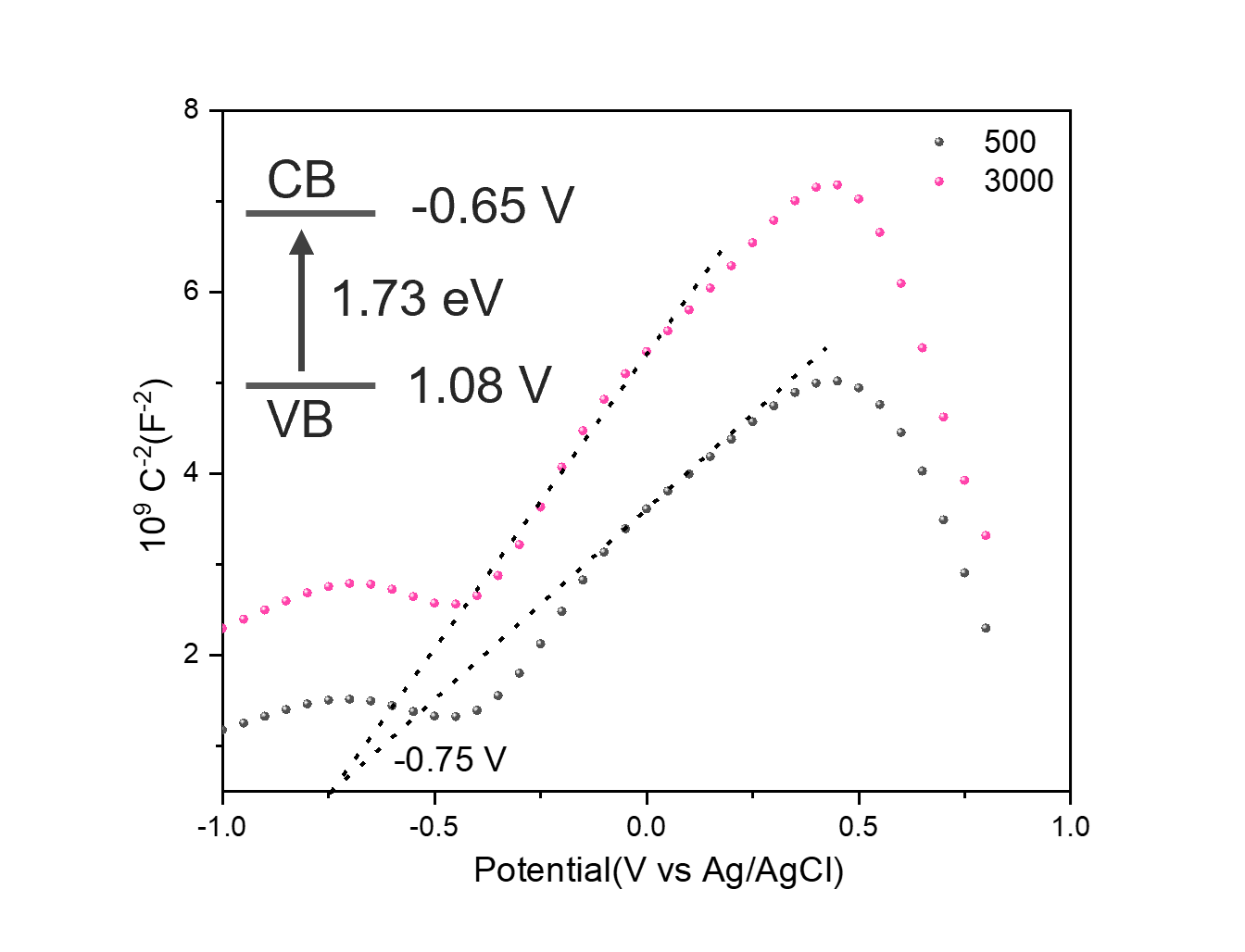


**Figure S11.** Mott-Schottky Plot of C_70_@COF.


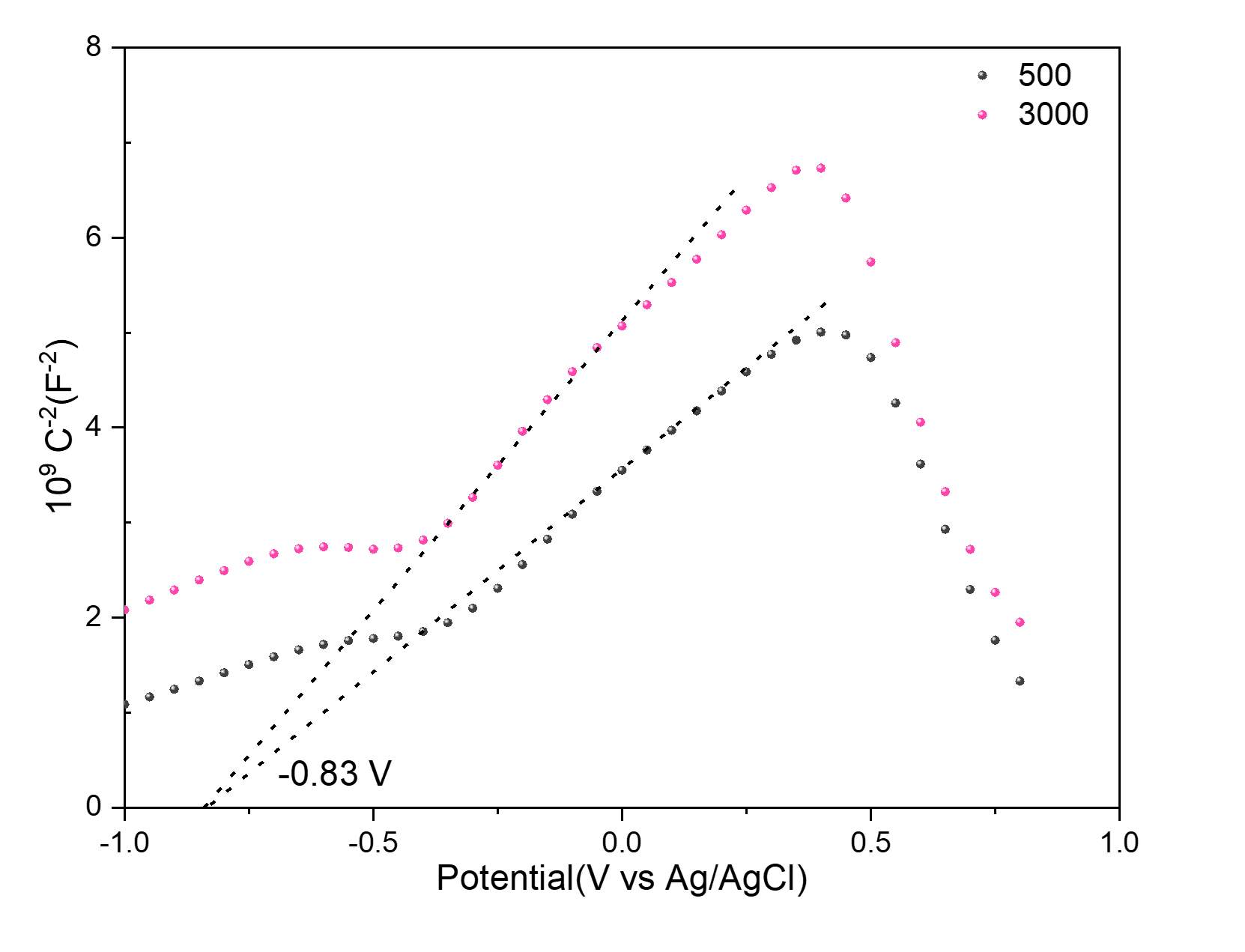


**Figure S12.** Mott-Schottky Plot of TTF-CoTPP COF.

3. Quantitative standard curve

**
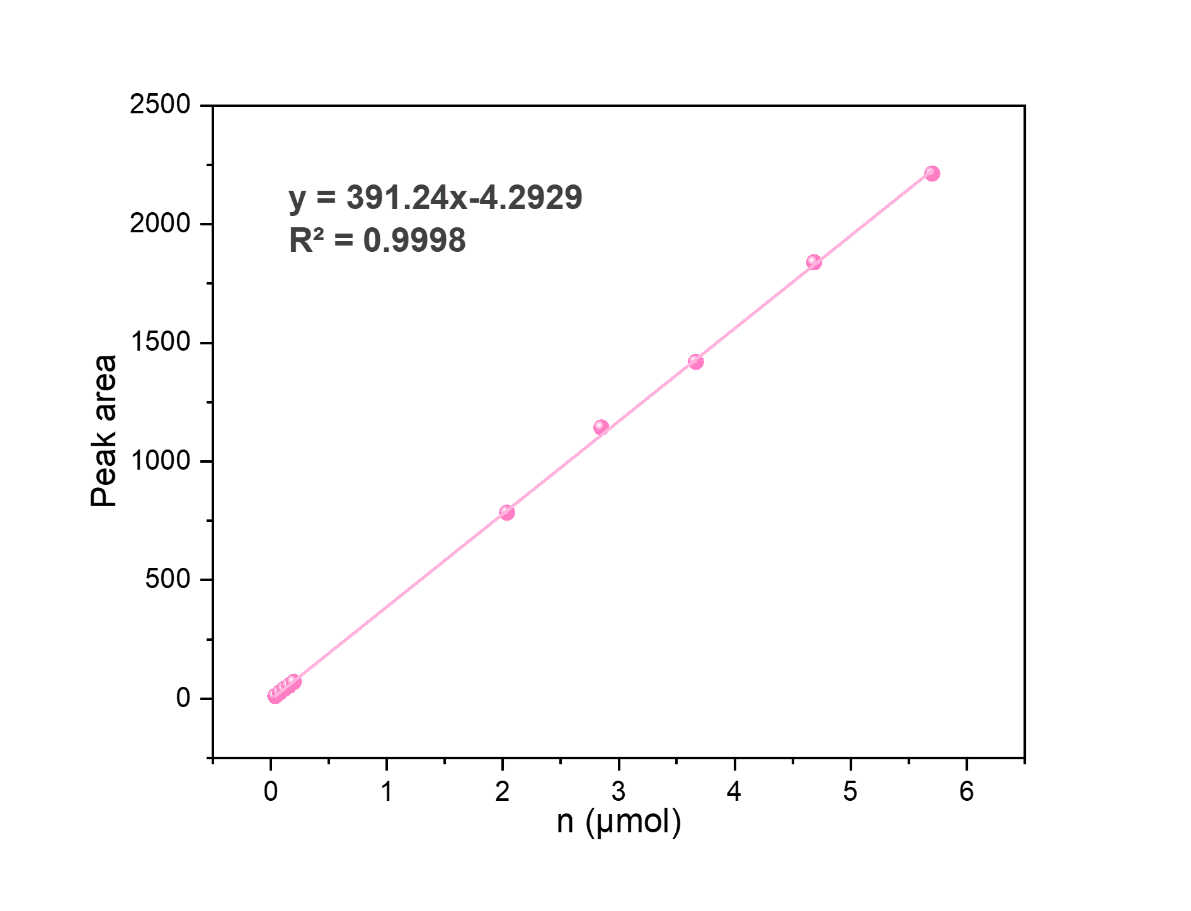
**

**Figure S13.** Quantitative standard curve for detection of CO production.

4. Photocatalytic efficiency


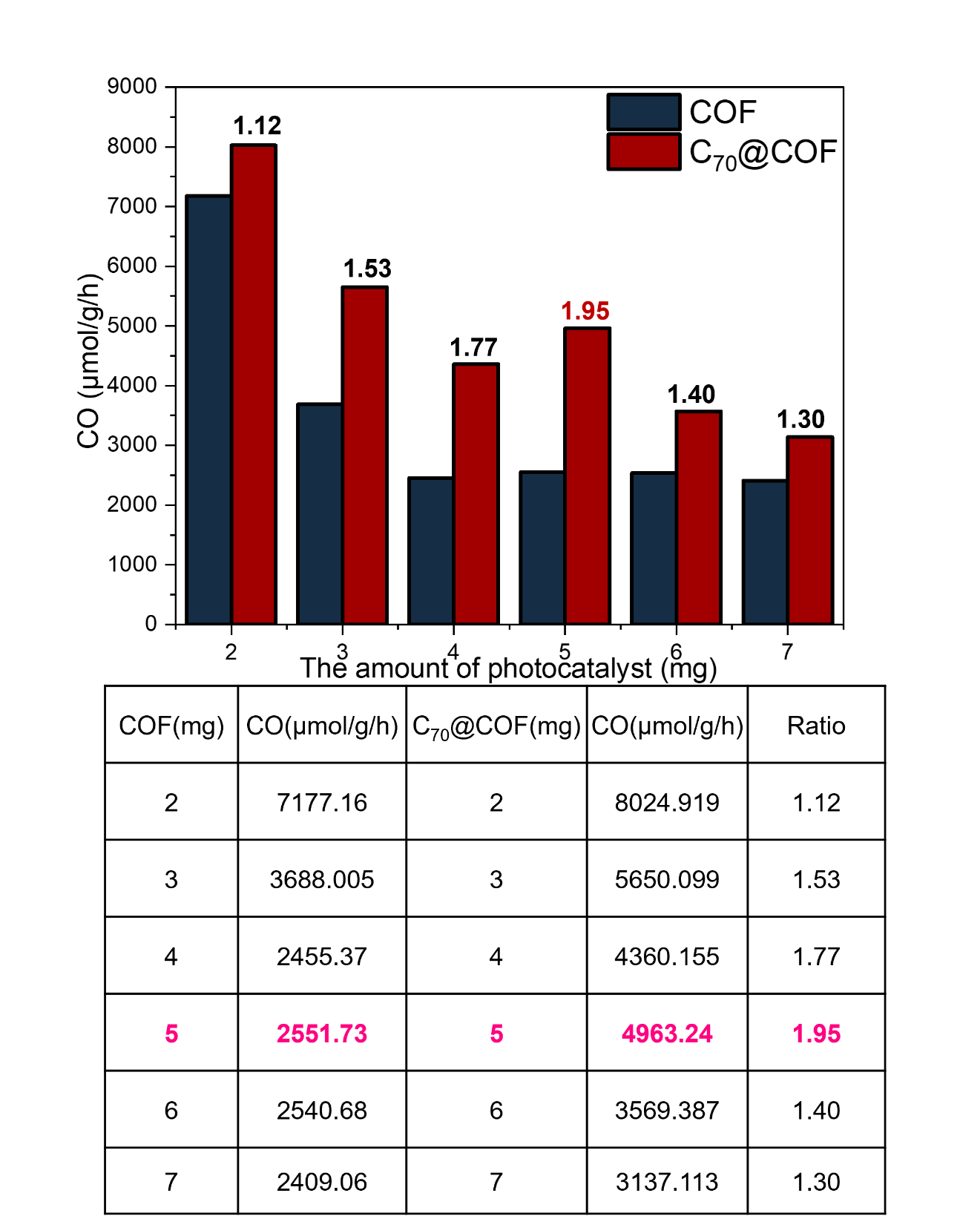


**Figure S14.** Comparison of photocatalytic efficiency of photocatalysts with different amounts.

The experimental procedure for photocatalytic CO_2_ reduction was as follows: varying amounts of the photocatalyst, along with 30 mg of Ru(bpy)_3_Cl_2_·6H_2_O and a mixed solution of acetonitrile, TEOA, and H_2_O in a 3:1:1 ratio (total volume of 15 mL), were sonicated uniformly and then added to a quartz reactor. The reactor was subsequently purged with high-purity CO_2_ for 30 minutes to completely eliminate oxygen. After 3 hours of light exposure, 1 mL gas was collected for testing.

The results indicated that using 2 or 3 mg of photocatalyst resulted in a high reduction rate; however, the incorporation of C_70_ did not significantly impact performance, likely due to the low loading amount of C_70_. In contrast, when 6 or 7 mg of photocatalyst was employed, the excessive presence of C_70_ hindered visible light absorption, leading to slight differences in performance. Based on these findings, 5 mg of photocatalyst was determined to be the optical amount, under which the reduction rate for C_70_@COF was found to be 1.95 times greater than that of pure COF, indicating that this specific amount balances light absorption and effective charge separation, thus maximizing the photocatalytic performance.


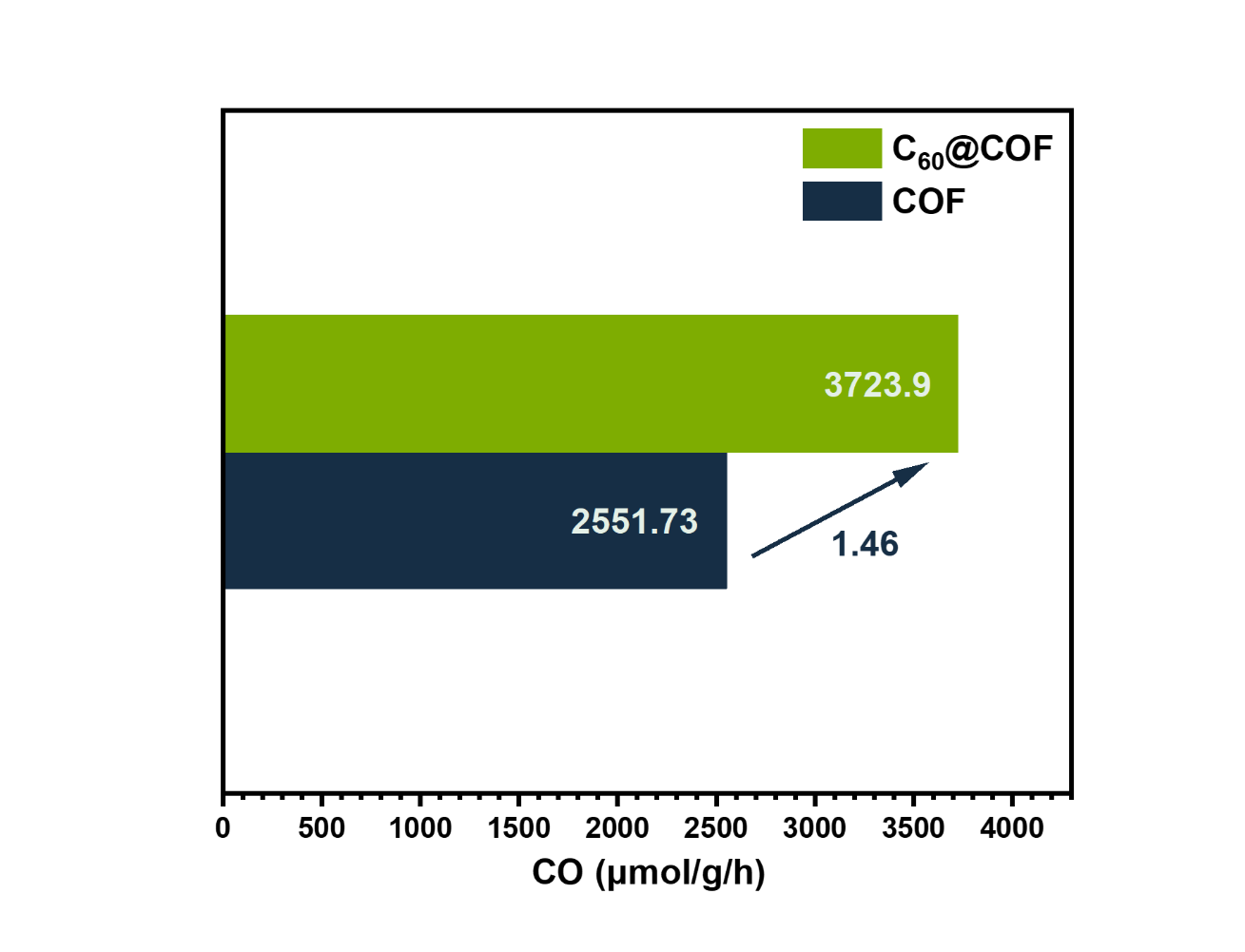


**Figure S15.** Photocatalytic CO₂ reduction performance of C_60_@CoTPP-TTF COF.

The synthesis and photocatalytic test of C_60_@CoTPP-TTF COF were performed following the same protocols as those established for C_70_@COF.


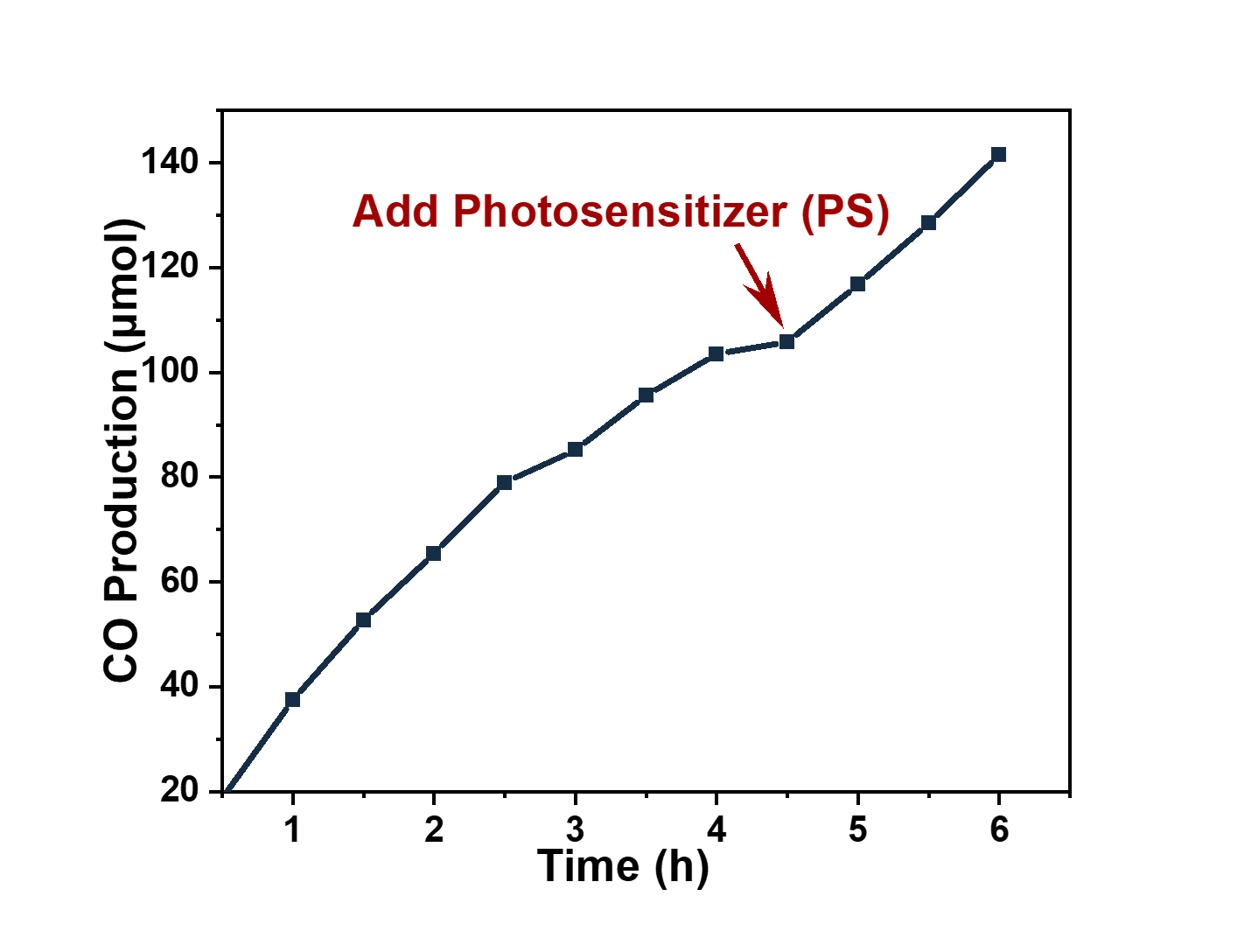


**Figure S16.** Time-dependent photocatalytic CO_2_ reduction performance of C_70_@COF.

This result reveals a decline in CO production after 3 hours of irradiation, attributed to photosensitizer deactivation. Upon reintroduced the photosensitizer, the CO production resumed rapidly.


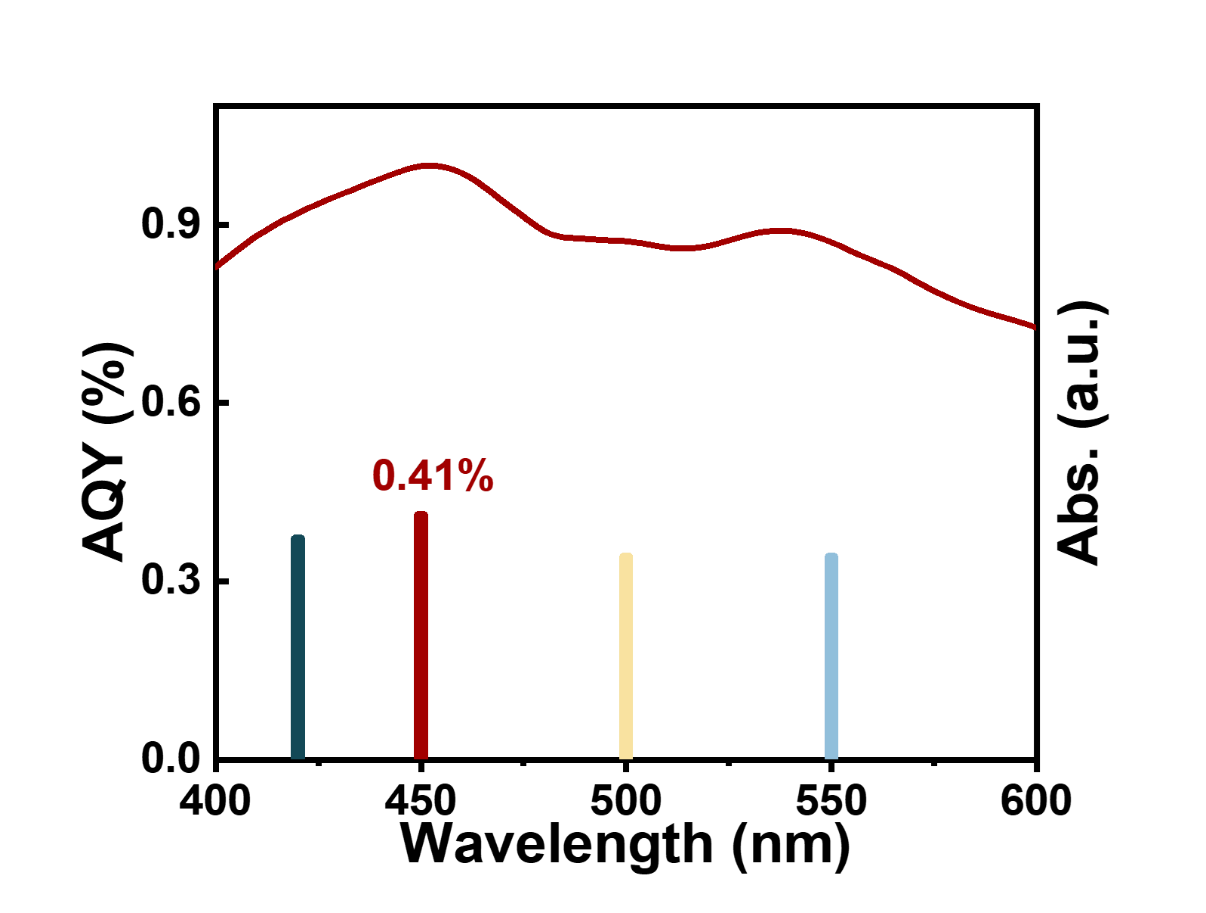


**Figure S17.** AQY of CO production catalyzed by C_70_@COF at different wavelengths and the related DRS.

5. Characterizations of photocatalyst after the reaction

**Figure S18.** XRD spectra of C_70_@COF before and after photocatalytic reaction.


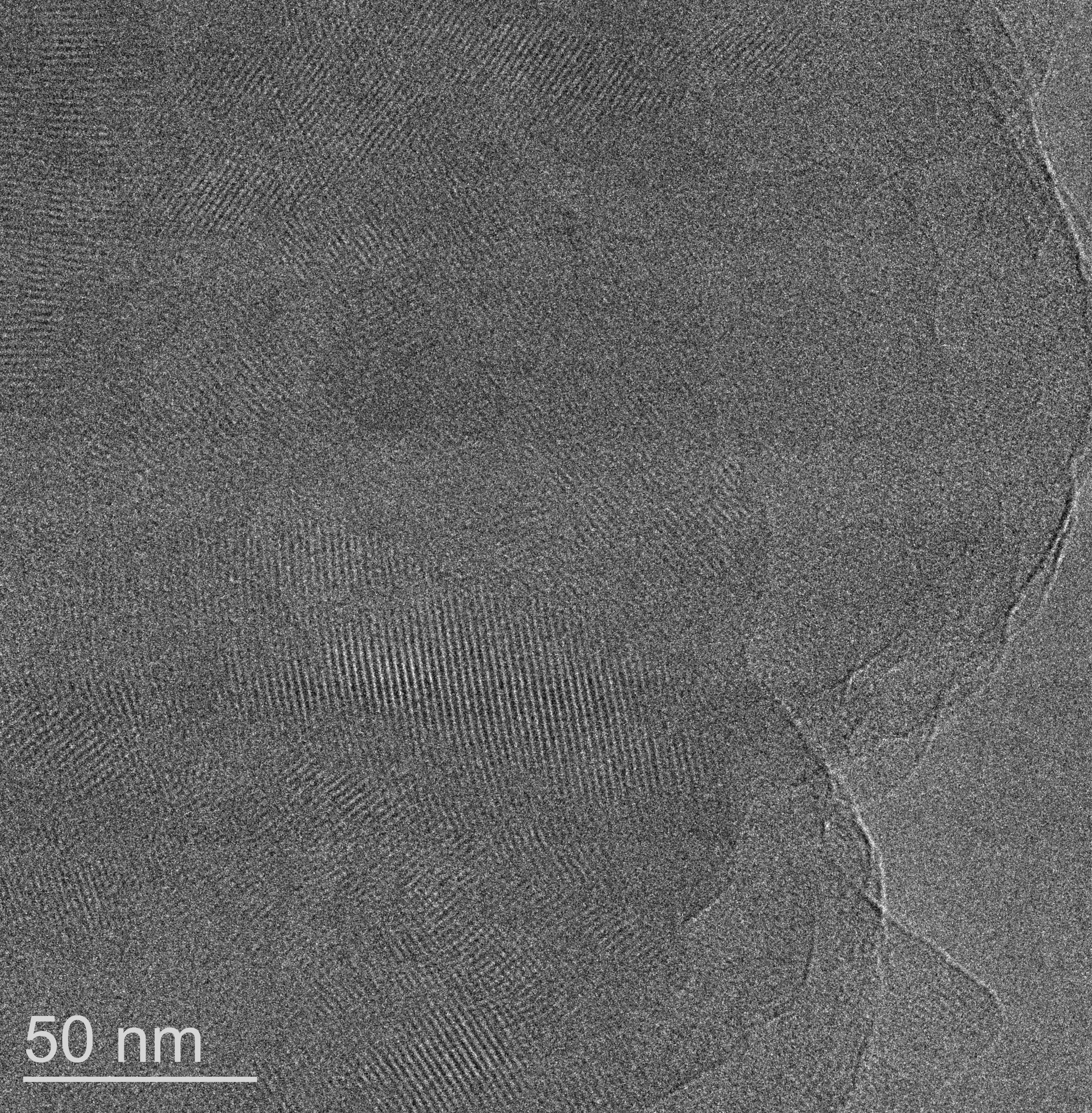


**Figure S19.** TEM image of C_70_@COF after photocatalytic reaction.

6. Time-resolved PL decay spectra


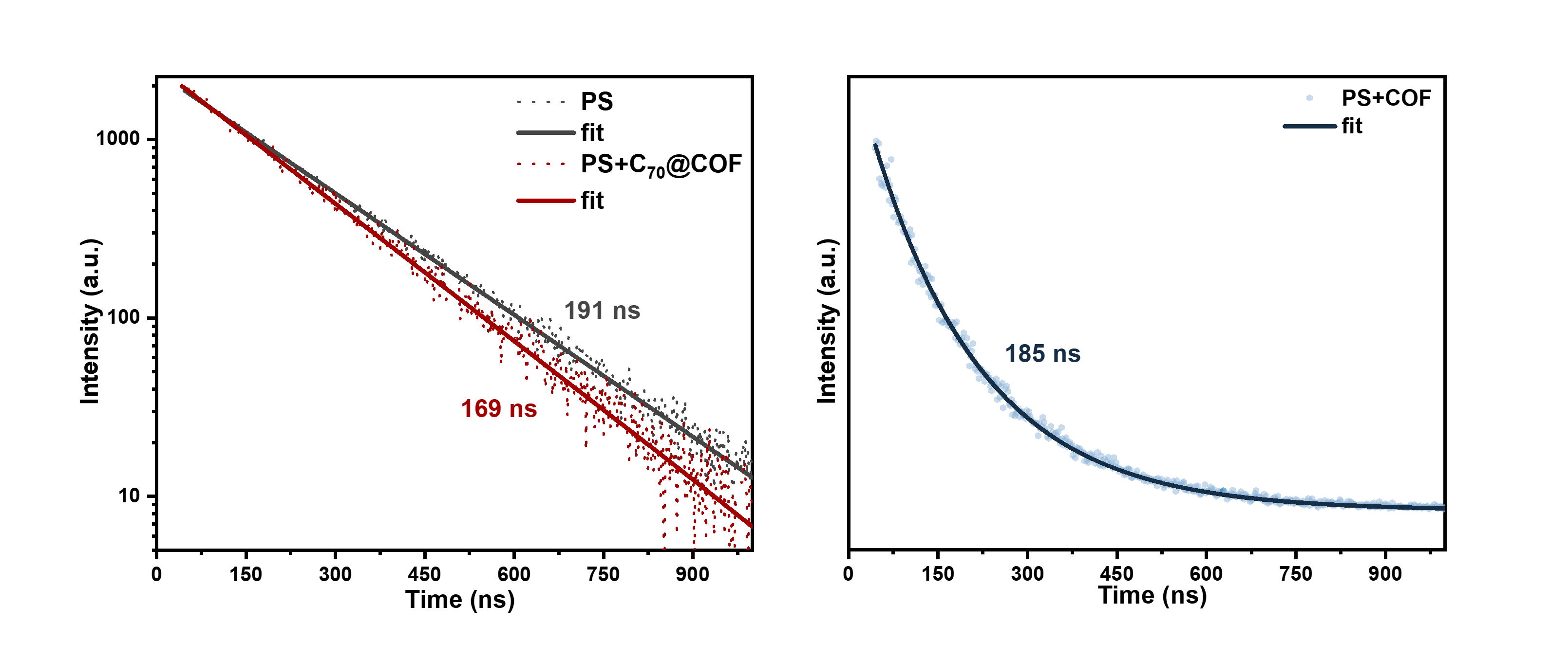


**Figure S20.** Time-resolved PL decay spectra of PS, PS+C_70_@COF and PS+COF, respectively.

7. Surface photovoltage response spectra


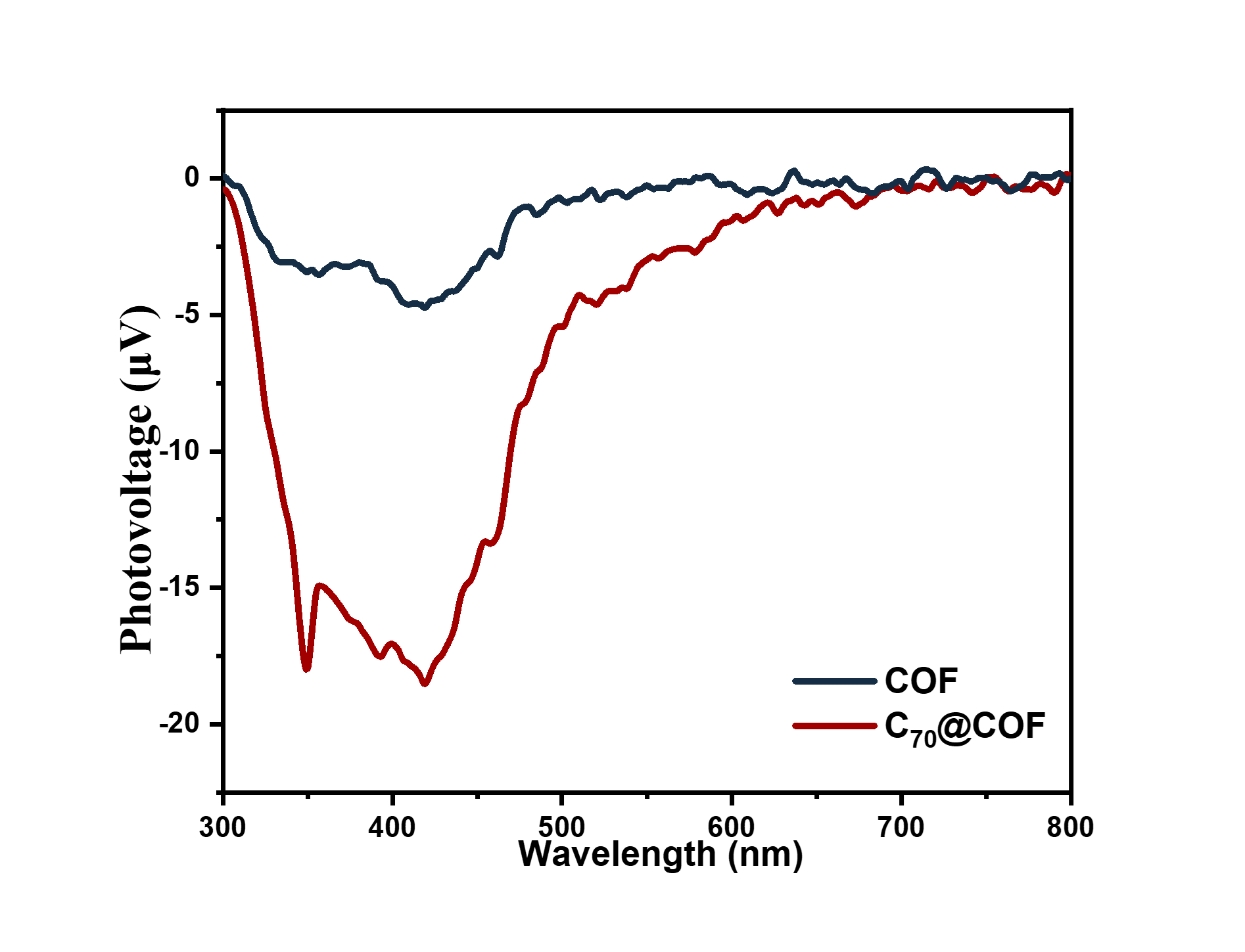


**Figure S21.** Surface photovoltage response (SPV) spectra of C_70_@COF and COF.

8. DFT Calculation


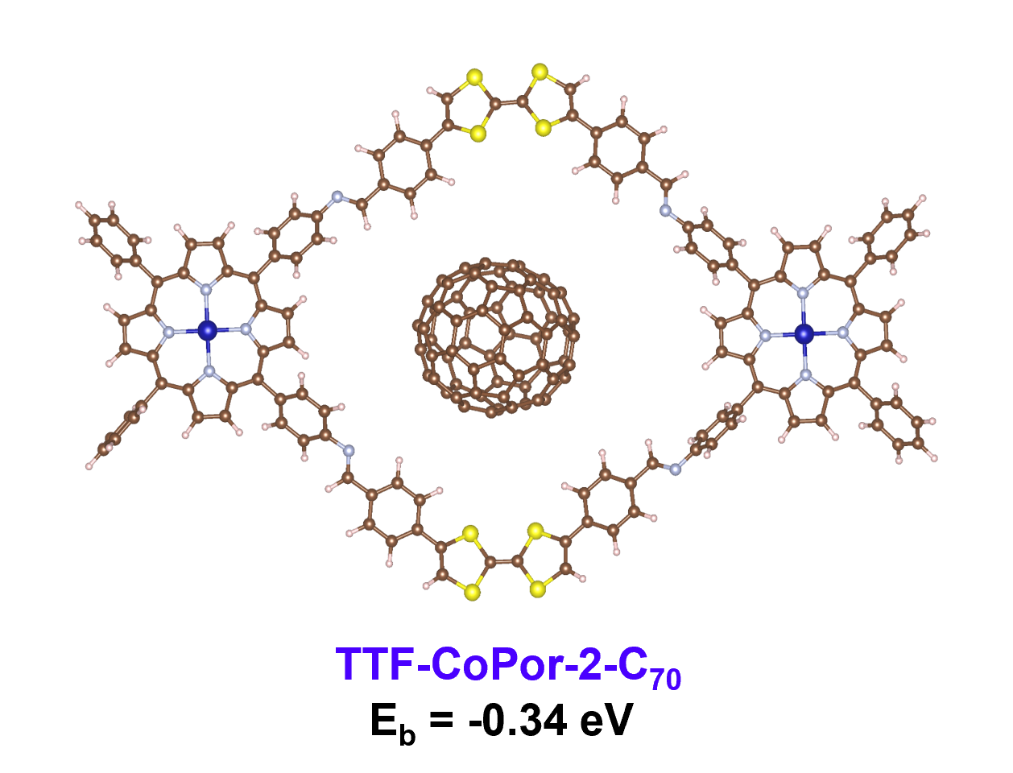


**Figure S22.** The binding energy for C_70_ encapsulated within TTF-CoPor-2.


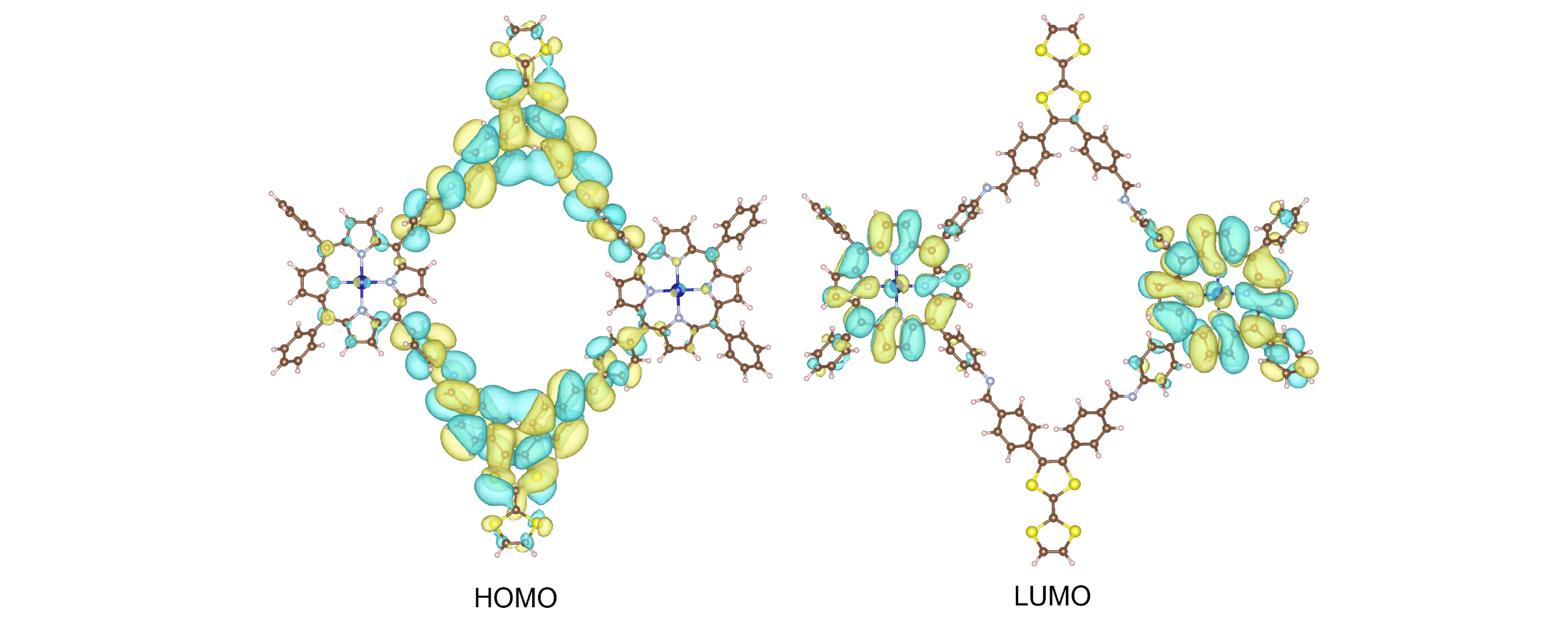


**Figure S23.** HOMO and LUMO distribution of COF.

9. Transient Absorption Spectra

**
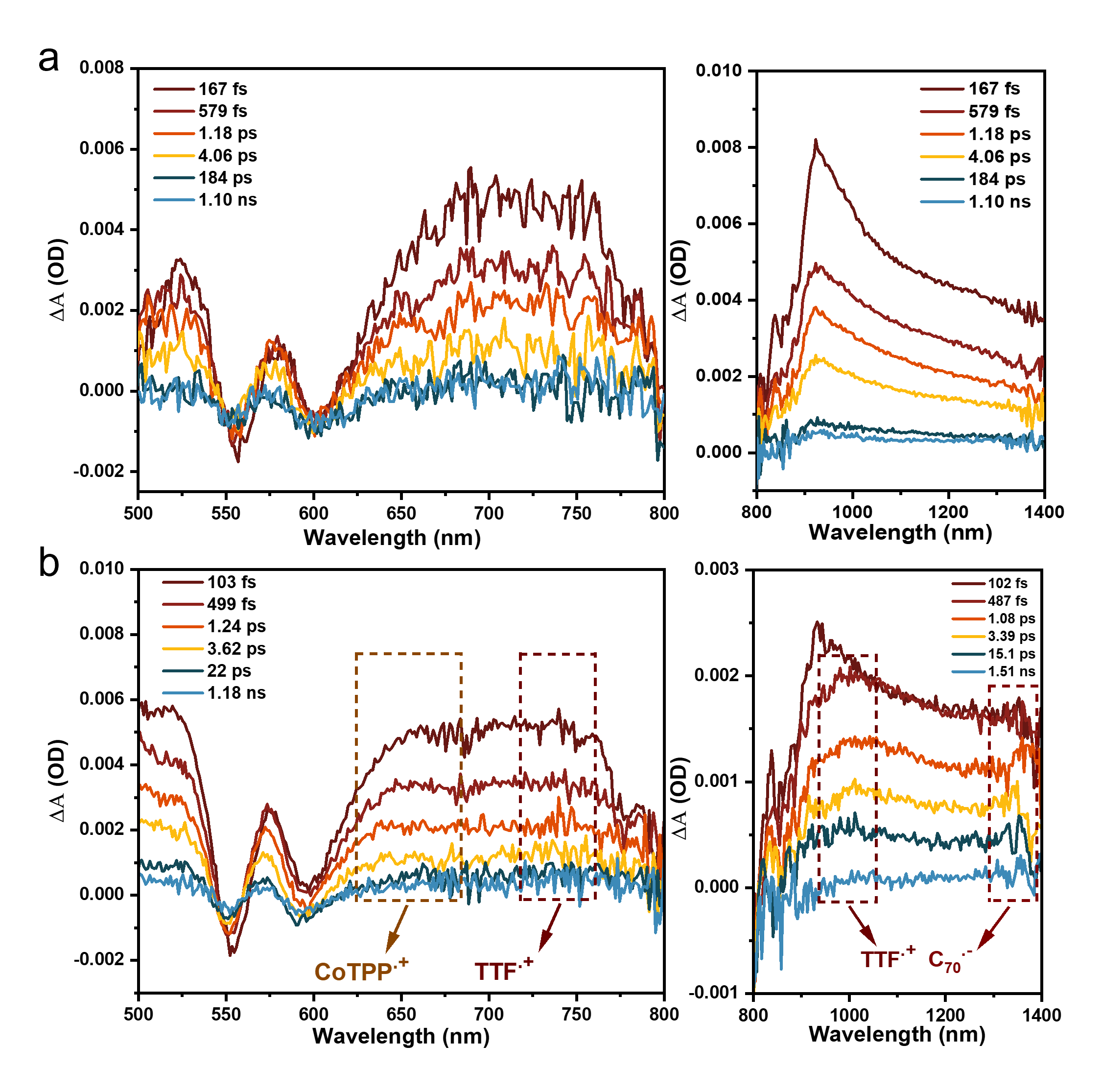
**

**Figure S24.** Selected TA spectrum of COF and C_70_@COF.

**
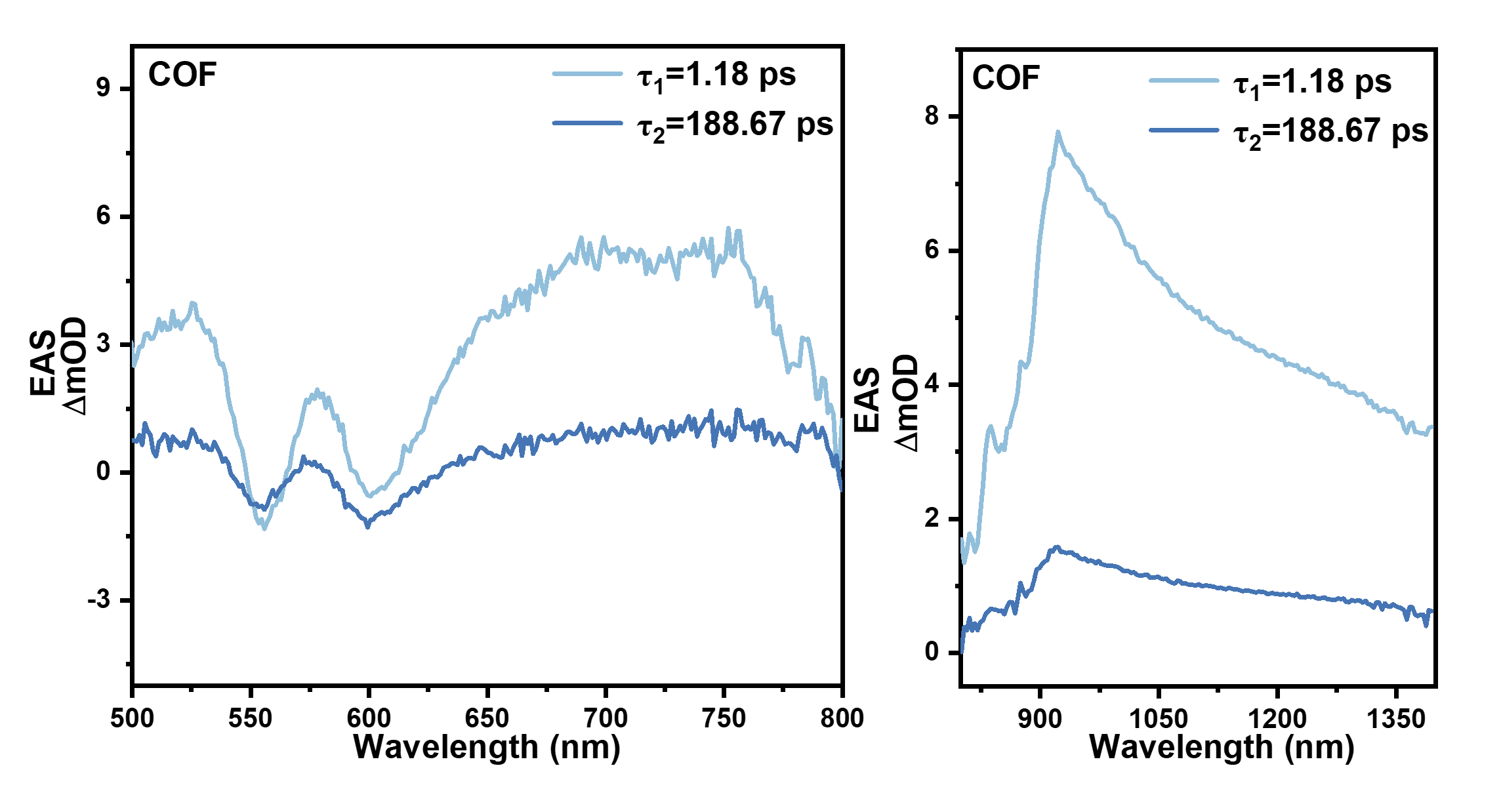
**

**Figure S25.** Evolution-associated spectra (EAS) of COF with negligible spectral evolution.

**
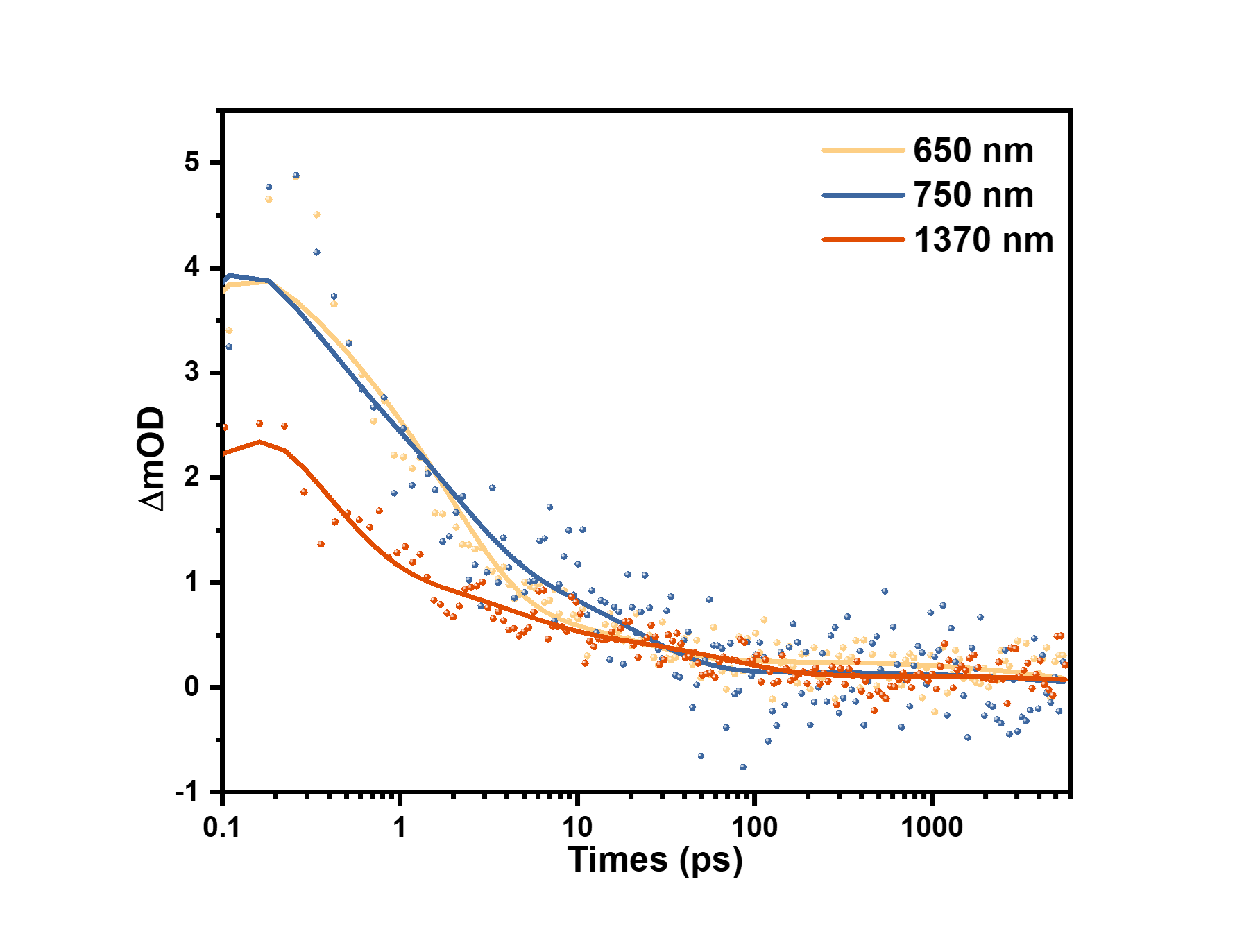
**

**Figure S26.** Normalized TA decay profile and fitting curves of C_70_@COF. Fitting kinetic curves are well matched with raw data at different wavelengths, showing the rationality of global analysis.

10. The intermediate states


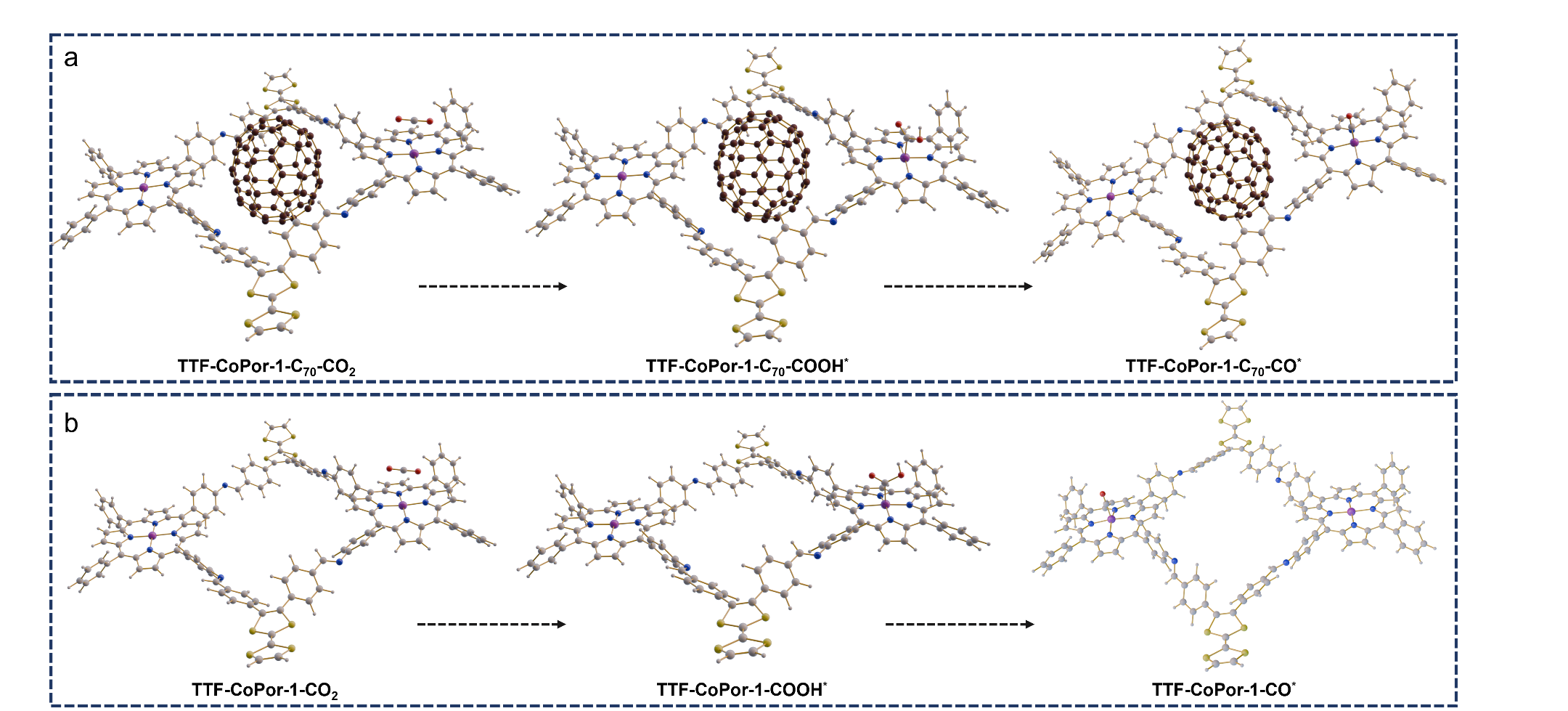


**Figure S27.** The intermediate states involved in the photochemical conversion from CO_2_ to CO. The elements of C, N, S, O, Co, H colored as gray, blue, yellow, red, purple, and light gray respectively.

**Table S1.** Summary of COF-based materials for photocatalytic CO_2_ reduction in recent years.

| **COFs** | **Photosensitizers/ Sacrificial agent** | **CO(μmol/g/h)** | **Ref** |
| --- | --- | --- | --- |
| **C_70_@TTF-CoTPP COF** | **[Ru(bpy)_3_]Cl_2_/ TEOA** | **4963.24** | **This work** |
| **TTF-CoTPP COF** | **[Ru(bpy)_3_]Cl_2_/ TEOA** | **2551.73** | **This work** |
| NiPor-BDOB | [Ru(bpy)_3_]Cl_2_/ TEA | 1770 | ^[7]^ |
| H-COF-Ni | [Ru(bpy)_3_]Cl_2_/ TEOA | 1958 | ^[8]^ |
| Ni-PCD@TD-COF | [Ru(bpy)_3_]Cl_2_/ TEOA | 480 | ^[9]^ |
| Ni-TpBpy-COF | [Ru(bpy)_3_]Cl_2_/ AA | 966 | ^[10]^ |
| NiP-TPE-COF | [Ru(bpy)_3_]Cl_2_/ TEOA | 525 | ^[11]^ |
| CoP-TPE-COF | [Ru(bpy)_3_]Cl_2_/ TEOA | 2410 | ^[11]^ |
| COF367-Co NSs | [Ru(bpy)_3_]Cl_2_/ AA | 10162 | ^[12]^ |
| DQTP-COF-Co | [Ru(bpy)_3_]Cl_2_/ TEOA | 1020 | ^[13]^ |
| Co/Cu_3_-TPA-COF | [Ru(bpy)_3_]Cl_2_/ TEOA | 13000 | ^[14]^ |
| Co-PyPor-COF | [Ru(bpy)_3_]Cl_2_/ TEOA | 9600 | ^[15]^ |
| Fe SAS/Tr-COFs | [Ru(bpy)_3_]Cl_2_/ TEOA | 980 | ^[16]^ |
| TFBD-COF-Co-SA | [Ru(bpy)_3_]Cl_2_/ TEOA | 1480 | ^[17]^ |
| Ni@TPHH-COF | [Ru(bpy)_3_]Cl_2_/ TEOA | 3300 | ^[18]^ |

# References

[1] J. VandeVondele, M. Krack, F. Mohamed, M. Parrinello, T. Chassaing, J. Hutter, *Comput. Phys. Commun.* **2005**, *167*, 103-128.

[2] S. Goedecker, M. Teter, J. Hutter, *Phys. Rev. B* **1996**, *54*, 1703-1710.

[3] C. Hartwigsen, S. Goedecker, J. Hutter, *Phys. Rev. B* **1998**, *58*, 3641-3662.

[4] J. VandeVondele, J. Hutter, *J. Chem. Phys.* **2007**, *127*, 114105.

[5] J. P. Perdew, K. Burke, M. Ernzerhof, *Phys. Rev. Lett.* **1996**, *77*, 3865-3868.

[6] S. Grimme, J. Antony, S. Ehrlich, H. Krieg, *J. Chem. Phys.* **2010**, *132*, 154104.

[7] S. Suleman, K. Sun, Y. Zhao, X. Guan, Z. Lin, Z. Meng, H.-L. Jiang, *CCS Chem.* **2024**, *6*, 1689-1697.

[8] S. Yang, R. Sa, H. Zhong, H. Lv, D. Yuan, R. Wang, *Adv. Funct. Mater.* **2022**, *32*, 2110694.

[9] H. Zhong, R. Sa, H. Lv, S. Yang, D. Yuan, X. Wang, R. Wang, *Adv. Funct. Mater.* **2020**, *30*, 2002654.

[10] W. Zhong, R. Sa, L. Li, Y. He, L. Li, J. Bi, Z. Zhuang, Y. Yu, Z. Zou, *J. Am. Chem. Soc.* **2019**, *141*, 7615-7621.

[11] H. Lv, R. Sa, P. Li, D. Yuan, X. Wang, R. Wang, *Sci. China Chem.* **2020**, *63*, 1289-1294.

[12] W. Liu, X. Li, C. Wang, H. Pan, W. Liu, K. Wang, Q. Zeng, R. Wang, J. Jiang, *J. Am. Chem. Soc.* **2019**, *141*, 17431-17440.

[13] M. Lu, Q. Li, J. Liu, F.-M. Zhang, L. Zhang, J.-L. Wang, Z.-H. Kang, Y.-Q. Lan, *Appl. Catal. B Environ.* **2019**, *254*, 624-633.

[14] X. Lan, H. Li, Y. Liu, Y. Zhang, T. Zhang, Y. Chen, *Angew. Chem. Int. Ed.* **2024**, e202407092.

[15] T.-X. Luan, J.-R. Wang, K. Li, H. Li, F. Nan, W. W. Yu, P.-Z. Li, *Small* **2023**, *19*, 2303324.

[16] L. Ran, Z. Li, B. Ran, J. Cao, Y. Zhao, T. Shao, Y. Song, M. K. H. Leung, L. Sun, J. Hou, *J. Am. Chem. Soc.* **2022**, *144*, 17097-17109.

[17] Y. Yang, Y. Lu, H.-Y. Zhang, Y. Wang, H.-L. Tang, X.-J. Sun, G. Zhang, F.-M. Zhang, *ACS Sustainable Chem. Eng.* **2021**, *9*, 13376-13384.

[18] M. Dong, J. Zhou, J. Zhong, H.-T. Li, C.-Y. Sun, Y.-D. Han, J.-N. Kou, Z.-H. Kang, X.-L. Wang, Z.-M. Su, *Adv. Funct. Mater.* **2022**, *32*, 2110136.
